# N6-Benzyladenosine Derivatives as Novel *N*-Donor Ligands of Platinum(II) Dichlorido Complexes

**DOI:** 10.3390/molecules18066990

**Published:** 2013-06-14

**Authors:** Pavel Štarha, Igor Popa, Zdeněk Trávníček, Ján Vančo

**Affiliations:** Regional Centre of Advanced Technologies and Materials, Department of Inorganic Chemistry, Faculty of Science, Palacký University, 17. listopadu 12, CZ-77146 Olomouc, Czech Republic; E-Mails: pavel.starha@upol.cz (P.S.); igor.popa@upol.cz (I.P.); jan.vanco@upol.cz (J.V.)

**Keywords:** platinum(II) complexes, transplatin derivatives, N6-benzyladenosine derivatives, multinuclear NMR, antitumor activity

## Abstract

The platinum(II) complexes *trans-*[PtCl_2_(L_n_)_2_]∙*x*Solv **1**–**13** (Solv = H_2_O or CH_3_OH), involving N6-benzyladenosine-based N-donor ligands, were synthesized; L_n_ stands for N6-(2-methoxybenzyl)adenosine (**L_1_**, involved in complex **1**), N6-(4-methoxy-benzyl)adenosine (**L_2_**, **2**), N6-(2-chlorobenzyl)adenosine (**L_3_**, **3**), N6-(4-chlorobenzyl)-adenosine (**L_4_**, **4**), N6-(2-hydroxybenzyl)adenosine (**L_5_**, **5**), N6-(3-hydroxybenzyl)-adenosine (**L_6_**, **6**), N6-(2-hydroxy-3-methoxybenzyl)adenosine (**L_7_**, **7**), N6-(4-fluoro-benzyl)adenosine (**L_8_**, **8**), N6-(4-methylbenzyl)adenosine (**L_9_**, **9**), 2-chloro-N6-(3-hydroxy-benzyl)adenosine (**L_10_**, **10**), 2-chloro-N6-(4-hydroxybenzyl)adenosine (**L_11_**, **11**), 2-chloro-N6-(2-hydroxy-3-methoxybenzyl)adenosine (**L_12_**, **12**) and 2-chloro-N6-(2-hydroxy-5-methylbenzyl)adenosine (**L_13_**, **13**). The compounds were characterized by elemental analysis, mass spectrometry, IR and multinuclear (^1^H-, ^13^C-, ^195^Pt- and ^15^N-) and two-dimensional NMR spectroscopy, which proved the N7-coordination mode of the appropriate N6-benzyladenosine derivative and *trans*-geometry of the title complexes. The complexes **1**–**13** were found to be non-toxic *in vitro* against two selected human cancer cell lines (HOS and MCF7; with IC_50_ > 50.0 µM). However, they were found (by ESI-MS study) to be able to interact with the physiological levels of the sulfur-containing biogenic biomolecule l-Methionine by a relatively simple 1:1 exchange mechanism (one L_n_ molecule was replaced by one l-Methionine molecule), thus forming a mixed-nitrogen/sulfur-ligand dichlorido-platinum(II) coordination species.

## 1. Introduction

Despite a formidable intensity of research and development in the field of metallopharmaceuticals, the group of platinum(II) complexes still leads in the number of treated patients, as well as in the number of marketed drugs and yearly sales [1]. The success of this group began with the discovery of anticancer activity of the *cis-*isomer of Peyrone’s salt [diammine-dichloridoplatinum(II)] [2], known as *cisplatin*, by the group of Rosenberg *et al*. [3], while the other, biologically nearly inactive *trans*-isomer (*transplatin*) remained just a scientific oddity for a long time. The conundrum of the inactivity of the *trans-*isomer was unravelled by the discoveries during the latest 20 years (for comprehensive reviews see [4,5] and the references therein), which showed that *trans-*platinum inspired complexes, such as those involving the aromatic heterocyclic ligands [6], aliphatic amines [7], or iminoether ligands [8], could exhibit cytotoxicity comparable to that of *cisplatin*, and also that some of these biologically active species could circumvent the mechanisms of resistance to *cisplatin* [9,10]. These discoveries fired up the recent development in the area and proved that *trans-*platinum complexes must be considered as biologically relevant, even if the mechanisms of their action are much more complex than in the case of *cisplatin*.

N6-benzyladenine and its substituted-benzyl derivatives represent the plant hormones from the group of aromatic cytokinins [11]. The free bases are responsible for their physiological action, while the ribosides, *i.e.*, N6-benzyladenosine and its substituted-benzyl derivatives, are one of the transport forms of the mentioned phytohormones. The quoted N6-benzyladenosines have been shown as *in vitro* antitumor active substances against various types of human cancer cell lines [12,13]. However, from the coordination chemistry point of view, only two papers dealing with the transition metal complexes with N6-benzyladenosine and its substituted-benzyl derivatives acting as N-donor ligands have been published to date. Concretely, Waysbort *et al*. studied the dinuclear rhenium acetato-bridged complexes with N6-benzyladenosine [14], while Trávníček et al. described a series of iron mixed-valence complexes involving N6-(substituted-benzyl)adenosine derivatives and their *in vitro* antitumor effect on four human cancer cell lines [15]. The [FeCl_3_(L_8_)_2_] complex, which involves N6-(4-fluorobenzyl)adenosine symbolized as **L_8_** in this work, was found to be moderately effective against human osteosarcoma (HOS; IC_50_ = 8.0 µM), human myelogenous leukemia (K562; IC_50_ = 9.0 µM) and human adenocarcinoma (MCF7; IC_50_ = 16.0 µM) cancer cells. These findings led us to study the utilization of *in vitro* anticancer active organic molecules (N6-benzyladenosine derivatives) as N-donor ligands of the coordination compounds of the biologically more perspective transition metal (*i.e.*, platinum). We chose thirteen synthetically relatively easily obtainable N6-benzyladenosine derivatives, which represent two types of compounds, *i.e.*, N6-(substituted-benzyl)adenosine derivatives (**L_1_**–**L_9_**) and 2-chloro-N6-(substituted-benzyl)adenosine derivatives (**L_10_**–**L_13_**), which act as N-donor ligands within the structures of *trans-*platinum(II) dichlorido complexes (**1**–**13**). The complexes were screened for their *in vitro* antitumor activity against two human cancer cell lines (HOS and MCF7) to find out whether the complex formation may enhance the cytotoxic effect of the ligands involved in the prepared complexes.

## 2. Results and Discussion

### 2.1. Characterizations and General Properties of the Complexes

The *trans-*[PtCl_2_(L_n_)_2_]∙*x*Solv complexes **1**–**13** (Solv = H_2_O or CH_3_OH) were prepared as white powders. Although, the preparation of *cis*-isomers was the main goal of this work, only the pure *trans*-isomers were isolated. This was quite surprising because two series of *cis*-[PtCl_2_(L)_2_] complexes with differently substituted N6-benzyladenine derivatives [16] or with 7-azaindole derivatives [17] were formerly prepared by the same procedure, *i.e.*, the reaction of K_2_[PtCl_4_] with the corresponding N-donor ligand. This unexpected behaviour may be connected with very low thermodynamic stability of the *cis*-isomers, which probably transform instantly into the *trans*-isomers. We tried to detect the *cis*-to-*trans* transformation process by means of ^195^Pt-NMR experiments, however, although the NMR spectra were measured immediately after the complex preparation, only the presence of pure *trans*-isomer was confirmed (see below for more details). 

**Scheme 1:**
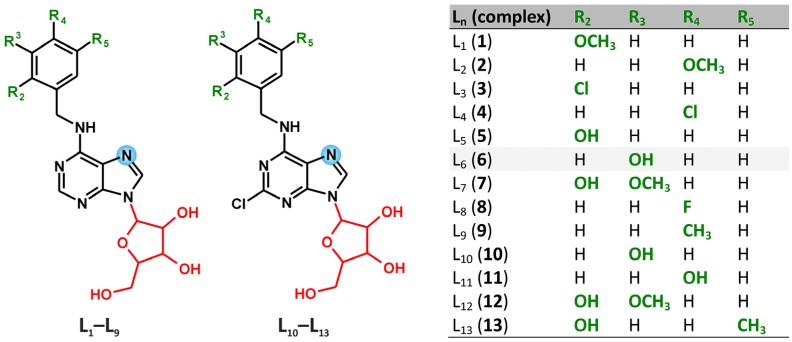
Schematic representations of the N6-benzyladenosine-based N-donor ligands (**L_1_**–**L_13_**) involved in structures of complexes **1**–**13**.

It has been found that the chlorination at the C2 atom on the purine moiety of the L_n_ ligand is important for final complex solubility, and thus the complexes **10**–**13**, which involve 2-chloro-N6-benzyladenosine derivatives, are more soluble in DMF, ethanol, methanol and acetone than complexes **1**–**9**, whose structures contain N6-benzyladenosines (see Scheme 1). A limited solubility in distilled water was observed for all the complexes. The obtained results of the elemental analysis for the prepared complexes are in good agreement with the calculated ones.

All the complexes behave as non-electrolytes in DMF solutions (1.2–3.5 S·cm^2^·mol^−1^) [18]. Thermal decomposition of **1**–**13** started right after the beginning of the experiment and is accompanied by a loss of the solvate molecules (½CH_3_OH for **1**, **5**, **8** and **9**, CH_3_OH for **2**, **4**, **6**, **7**, **11** and **12**, ½H_2_O for **3** and ¾CH_3_OH for **10** and **13**). The calculated weight losses of the desolvation process differ from the calculated ones by no more than 0.3%. The desolvated complexes decay to PtO without the formation of any thermally stable intermediates from 100–186 °C to 476–595 °C. 

The ESI– mass spectra of **1**–**13** dissolved in methanol were found to be similar in terms of the detected types of species (see Supplementary Material). The pseudomolecular peaks corresponding to [PtCl_2_(L_n_)_2_-H]^−^ were detected in the ESI– mass spectra of **1**–**13**. Typically, the most intense peaks belonged to the [PtCl_3_(L_n_)_2_]^−^ species. The species whose composition corresponded to the other detected peaks, involved the appropriate adenine derivative without ribose (symbolized as L_n_`). Concretely [PtCl_2_(L_n_)(L_n_`)-H]^−^, [PtCl(L_n_)(L_n_`)-2H]^−^ and [PtCl(L_n_`)-2H]^−^ were found in the spectra.

Detection of the characteristic bands of the appropriate N6-benzyladenosine derivatives (**L_1_**–**L_13_**; [19,20]) in the IR spectra of the complexes **1**–**13** proved the presence of the mentioned organic ligands in the structure of the studied complexes. The bands were found and assigned as follows: the medium to strong bands at 3408–3584 cm^−1^ can be attributed to *ν*(O–H) of the ribose moiety, the medium to strong bands between 3308 and 3359 cm^−1^ are assignable to *ν*(N–H) and the medium bands between 3121 and 3133 cm^−1^ belong to *ν*(C–H)_ar_. It should be pointed out that the interpretation of the bands in the region of 3100–3600 cm^−1^ is markedly influenced by overlapping of the vibrations. The majority of the bands found in the 660–900 cm^−1^ and 1450–1590 cm^−1^ region are assignable to the skeletal vibrations of the purine moiety, while the bands at 1605–1616 cm^−1^ can be attributed *ν*(C=N)_ar_ purine skeletal vibrations [20]. The substitutions at the aromatic rings were proved by the presence of the stretching vibrations of *ν*(C–Cl)_ar_ (**3**, **4**, **11**–**13**), *ν*(C–F)_ar_ (**8**) and *ν*(C–O)_ar_ (**1**, **2**, **5**–**7**, **11**–**13**) in the regions of 1152–1182, at 1215, and 1214–1248 cm^−1^, respectively, in the IR spectra. The bands of medium to strong intensity at 1053–1116 cm^−1^ can be attributed to the *ν*(C–O)_aliph_ vibrations, which belong to the hemiacetal groups of the ribose moiety. The bands between 326 and 349 cm^−1^ are assignable to *ν*(Pt–Cl), while those detected between 510 and 545 cm^−1^ belong to *ν*(Pt–N) [21].

**Figure 1:**
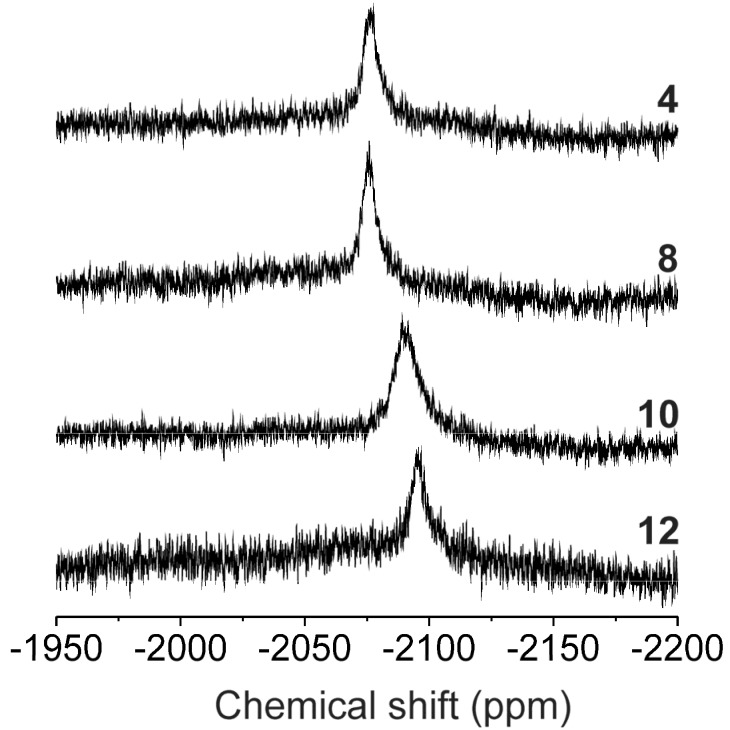
^195^Pt-NMR spectra of the selected complexes **4** and **8** (involving N6-benzyladenosine derivatives) and **10** and **12** (involving 2-chloro-N6-benzyladenosine derivatives).

The ^1^H-, ^13^C- and ^15^N-NMR signals of free N6-benzyladenosines **L_1_**–**L_13_** were detected in the spectra of the complexes (except for N3 in **13**). The ^1^H-, ^13^C- and ^15^N-NMR coordination shift values (Δ*δ* = *δ_complex_* − *δ_ligand_*, ppm; see Table 1) clearly showed the N7-coordination mode of the **L_1_**–**L_13_** molecules to the central Pt(II) atom, similarly as reported for the platinum(II) dichlorido complexes involving N9-substituted N6-benzyladenine (analogous NMR studies in combination with crystallography; [16]) or adenine [22] derivatives. The greatest coordination shifts in the ^1^H-NMR and ^13^C-NMR spectra were found for the atoms situated close to the coordination site represented by the N7 atom, *i.e.* N6H, C8H, C5 and C8 (Table 1). The ^15^N signals found by ^1^H–^15^N gs-HMBC experiments proved beyond all doubt the coordination via the N7 atom. It is obvious that the signals of the N7 atom are shifted much more as compared with other nitrogen atoms involved in the structure of the studied complexes (Table 1) that means the N1, N3, N6 and N9 atoms are not significantly influenced by the ligand coordination to Pt(II) atom. It is well known that conclusions about the isomer types can be obtained from ^195^Pt-NMR experiments, where significant differences between chemical shifts of *cis-* (*δ^cis^*) and *trans-* (*δ^trans^*) complexes are expected, as was already proved in our previous papers regarding NMR and crystallographic study of the *cis*-to-*trans* isomerisation of platinum(II) dichlorido complexes with differently substituted N6-benzyladenine derivatives, with *δ^cis^* ≈ −2020 ppm and *δ^trans^* ≈ −2065 ppm [16]. As can be seen from the chemical shifts listed in Table 1 and depicted in Figure 1, those ones assignable to *trans*-isomers **1**–**13** correlated well with the previously reported values. Moreover, the number, position and intensity of the signals detected in the ^195^Pt-NMR spectra of **1**–**13** did not change in time (up to eight weeks), which is typical feature of *trans*-isomers of platinum(II) dichlorido complexes, while being highly improbable for the *cis*-isomers.

**Table 1:** The ^1^H-, ^13^C- and ^15^N-NMR coordination shifts (∆*δ* = *δ_complex_* − *δ_ligand_*; ppm) of the selected atoms and ^195^Pt chemical shifts (*δ*; ppm) of the prepared complexes.

	**^1^H-NMR**			**^13^C-NMR**					**^15^N-NMR**					**^195^Pt**
	C2H	N6H	C8H	C2	C4	C5	C6	C8	N1	N3	N6	N7	N9	
**1**	−0.09	0.82	0.84	1.35	−0.82	−4.07	−2.36	2.76	3.82	1.17	8.47	−105.03	5.60	−2077.10
**2**	0.18	0.52	0.84	1.41	−0.85	−4.03	−2.28	2.92	5.03	2.00	6.67	−105.02	6.17	−2072.08
**3**	0.17	0.60	0.88	1.37	−0.80	−4.01	−2.27	2.96	3.24	0.82	6.57	−106.00	4.96	−2074.81
**4**	0.19	0.51	0.92	1.27	−0.98	−4.14	−2.37	2.88	3.61	1.55	4.96	−106.69	5.45	−2071.38
**5**	0.12	0.69	0.72	1.61	−0.82	−3.90	−1.91	2.62	7.11	0.70	5.05	−105.70	5.09	−2076.80
**6**	0.18	0.59	0.84	1.31	−0.88	−4.07	−2.29	2.93	3.85	1.91	5.51	−105.78	6.21	−2074.68
**7**	0.15	0.75	0.69	1.49	−0.92	−3.97	−4.06	2.73	6.98	0.34	5.82	−106.22	4.78	−2078.30
**8**	0.19	0.52	0.90	1.31	−0.93	−4.13	−2.29	2.90	3.67	0.33	6.52	−105.37	6.32	−2071.68
**9**	0.19	0.55	0.83	1.34	−0.88	−4.07	−2.32	2.89	4.46	1.76	4.89	−106.10	5.86	−2073.15
**10**	−	0.26	0.83	−0.53	−1.19	−3.76	−0.66	3.03	3.13	−0.31	6.15	−106.52	5.02	−2084.94
**11**	−	0.60	0.83	1.30	−1.01	−3.68	−2.18	3.14	3.45	0.26	3.30	−106.24	5.00	−2083.47
**12**	−	0.81	0.69	1.34	−0.90	−3.66	−2.31	3.04	3.92	0.16	4.90	−105.72	5.03	−2088.28
**13**	−	0.85	0.78	0.12	−0.83	−3.27	−0.60	1.81	4.89	–	4.96	−105.76	5.33	−2091.62

### 2.2. *In Vitro* Cytotoxic Activity

The *trans-*complexes **1**–**13** were screened for their *in vitro* antitumor effect against two human cancer cell lines, namely breast adenocarcinoma (MCF7) and osteosarcoma (HOS). We also tested K_2_[PtCl_4_] and *cisplatin* to compare potential antitumor activity of the prepared complexes with the precursors and with commercially used platinum-based drug (N6-benzyladenosine derivatives involved in the structures of **1**–**13** were not tested, since their *in vitro* antitumor activity against HOS and MCF7 was previously reported [12]). Unfortunately, although the complexes **1**–**13** were found to be well soluble (>50.0 µM) in the medium used (0.1% DMF in distilled water), in other words they should be quite bioavailable, they did not show any cytotoxic effects up to the highest concentration tested (50.0 µM concentration, IC_50_ > 50.0 µM). As for K_2_[PtCl_4_] it was found to be inactive up to 50.0 µM. The IC_50_ values determined for *cisplatin* equal 18.1 ± 5.1 µM (MCF7) and 25.4 ± 8.5 µM (HOS).

Although the results of *in vitro* antitumor activity of **1**–**13** against HOS and MCF7 do not seem promising, it should be noted that situation regarding the free ligands L_n_ is completely different as shown in a Czech patent [12], where it is demonstrated that some of the ligands **L_1_**–**L_12_** (**L_13_** was not reported) are moderately to highly effective against HOS (IC_50_ = 2.8 µM for **L_5_**, 20.0 µM for **L_8_**, 21.2 µM for **L_1_**, >166.7 µM for **L_2–4,6,7,9–12_**), MCF7 (IC_50_ = 11.4 µM for **L_5_**, 14.0 µM for **L_8_**, 23.0 µM for **L_12_**, 27.0 µM for **L_7_**, >166.7 µM for **L_1–4,6,9–11_**), and human T-lymphoblastic (CEM; IC_50_ = 3.2 µM for **L_1_**, 14.5 µM for **L_3_**, 10.2 µM for **L_4_**, 0.7 µM for **L_5_**, 51.9 µM for **L_6_**, 0.3 µM for **L_7_**, 1.3 µM for **L_8_**, 47.8 µM for **L_10_**, 39.7 µM for **L_11_**, 0.2 µM for **L_12_**) and promyelocytic (HL-60; IC_50_ = 2.3 µM for **L_1_**, 1.6 µM for **L_3_**, 1.7 µM for **L_4_**, 0.4 µM for **L_5_**, 23.7 µM for **L_6_**, 0.2 µM for **L_7_**, 1.2 µM for **L_8_**, 15.9 µM for **L_10_**, 9.5 µM for **L_11_**, 0.1 µM for **L_12_**) leukemic cell lines. As already mentioned in the Introduction, one of the main reasons for the present study was to utilize these organic molecules as N-donor ligands in platinum(II) complexes and find out whether the complex formation may influence the resulting cytotoxic effect of the prepared complexes. Therefore, two human cancer cell lines (HOS and MCF7) were chosen for a biological screening. Unfortunately, it has been found that incorporation of the N6-benzyladenosine derivatives **L_1_**–**L_13_** into *trans*-platinum(II) dichlorido complexes **1**–**13** did not produce the expected goal. However, it is possible that the prepared complexes may show a selective cytotoxicity against human cancer cell lines and thus, next time-consuming experiments on diverse types of human cancer cell lines will be necessary to prove this presumption. Anyway, it is also known that initially inactive platinum complexes may serve as pro-drugs [23,24] owing to their metabolic activation, *e.g*. by interactions with diverse biomolecules such as reducing amino acids or proteins.

### 2.3. Interactions of the Selected Complexes ***7*** and ***12*** with l-Methionine, Evaluated by Mass Spectrometry

In an effort to identify the actual active species which might be biologically relevant in terms of long-lasting biological activity (due to anticipated slow kinetics of L_n_-**Pt(II)-S**-Met interaction) and to mechanistically describe the basic modes of interactions of two representative complexes **7** and **12** with relevant sulfur-containing biomolecule (l-Methionine), the electrospray-ionization mass-spectrometry (ESI-MS) experiments were carried out in both negative and positive ionization modes. To simulate at least one aspect of the physiological conditions, the solution of l-Methionine was used containing the physiological levels (20 μM) of this significant sulfur-containing biomolecule [25]. 

The corresponding interaction systems were formed by mixing the two freshly prepared working solutions: (solution 1) containing l-Methionine (at the final concentration of 20 μM) and (solution 2) containing the solutions of **7** or **12** in methanol, to give the final concentration of 10 μM (the final ratio of H_2_O:MeOH was 1:1 v/v). The resulting interacting solutions were measured immediately after the preparation and 12 h after preparation either. The interacting systems stayed clear during the whole 12 h of the observation.

**Figure 2:**
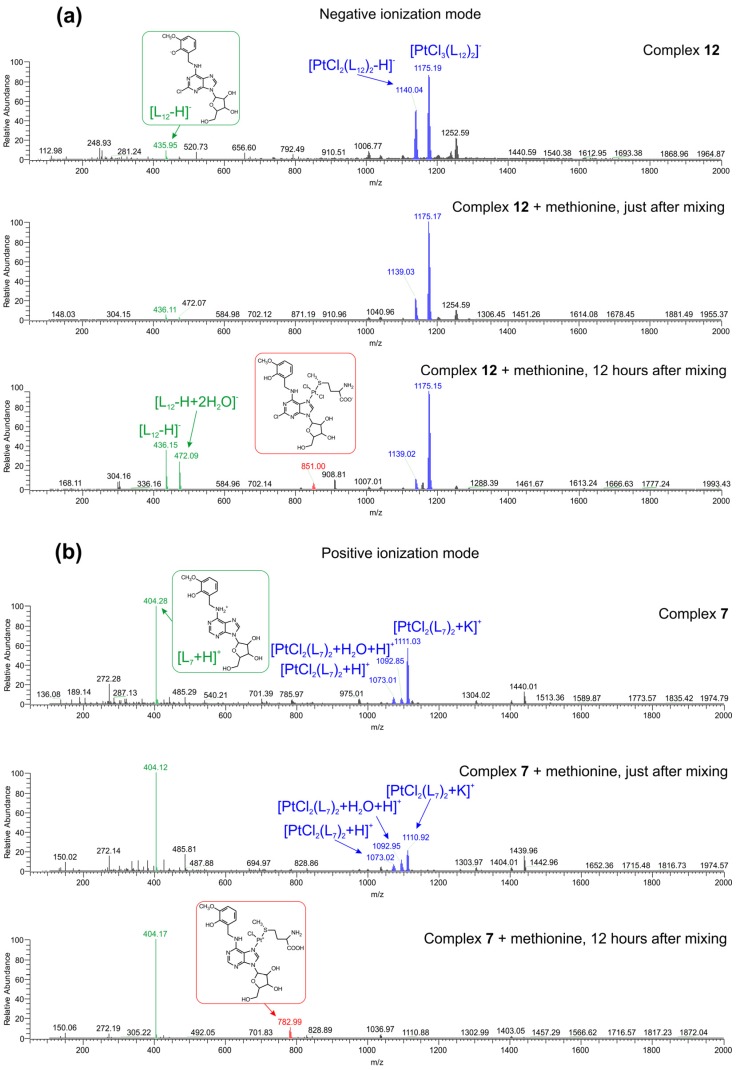
(**a**) The ESI− mass spectra of **12** (top spectrum), and interacting systems with l-Methionine measured immediately after the preparation (middle spectrum) and 12 h after the preparation (lower spectrum); (**b**) the ESI+ mass spectra of **7** (top spectrum), and interacting systems with l-Methionine measured immediately after the preparation (middle spectrum) and 12 h after the preparation (lower spectrum).

The ESI-MS experiments revealed a relatively simple 1:1 exchange mechanism (one L_n_ molecule was replaced by one l-Methionine molecule) in the measured interacting systems (Figure 2). In the control samples, composed only by the solutions of the complexes **7**, and **12**, respectively, the following main ions were identified in the negative ionization mode for **12** (see Figure 2a), top spectrum; *m/z*, [the corresponding pseudomolecular ion]^−^, sorted by the decreasing relative abundance]: 1175.19, [PtCl_3_(L_12_)_2_]^−^; 1140.04, [PtCl_2_(L_12_)_2_-H]^−^; 435.95, [L_12_-H]^−^; and in positive ionization mode for **7** (see Figure 2b), top spectrum; *m/z*, [the corresponding pseudomolecular ion]^+^, sorted by the decreasing relative abundance]: 404.28, [L_7_+H]^+^; 1111.03, [PtCl_2_(L_7_)_2_+K]^+^; 1073.01, [PtCl_2_(L_7_)_2_+H]^+^; 1092.85, [PtCl_2_(L_7_)_2_+H+H_2_O]^+^. An analogous set of pseudomolecular ions, as presented in Figure 2, was identified in the corresponding mass spectra of **7** measured in the negative ionization mode and **12** measured in the positive ionization mode (Figure S1). No significant qualitative differences, apart from the appearance of pseudomolecular ions (see Figure 2a), middle spectrum; *m/z*, [the corresponding pseudomolecular ion]^−^]: 148.03 [(L-Met)-H]^−^; and Figure 2b, middle spectrum; *m/z*, [the corresponding pseudomolecular ion]^+^): 150.02 [(L-Met)+H]^+^ corresponding to the presence of l-Methionine in the interacting systems, were observed in the measured mass spectra just after mixing the solutions of **7** and **12** with l-Methionine in both the negative and positive modes of electrospray-ionization. 

In accordance with the established slower kinetics of interactions between the dichlorido-platinum(II) complexes involving the N-donor co-ligands and methionine [26], we identified the significant changes in both qualitative and quantitative aspects of the mass spectra of the interacting systems measured 12 h after the mixing of their principal components. The mass spectra measured in both ionization modes (see Figure 2a,b), lower spectra, proved the formation of the analogous coordination species—see Figure 2a, lower spectrum; *m/z*, [the corresponding pseudomolecular ion]^−^): 851.00 [PtCl_2_(L_12_)(L-Met)-H]^−^; and Figure 2b, lower spectrum; *m/z*, [the corresponding pseudomolecular ion]^+^): 782.99 [PtCl(L_7_)(L-Met)]^+^], a (di)chlorido-platinum(II) complex containing one ligand of adenosine derivative and one l-Methionine ligand in its coordination sphere. The corresponding cleavage of one adenosine ligand from **7**, and **12**, respectively, manifested itself quantitatively by the increased ratio between the relative abundance of pseudomolecular ions of the free adenosine ligands and the relative abundance of pseudomolecular ions of **7** or **12**.

As some of the *transplatin* derived complexes involving the sulfur-containing ligands proved their remarkable cytotoxic effects [27], the obtained results of interaction studies suggest that the slow kinetics of the ligand exchange between the studied complexes and sulfur-containing biomolecules might represent the essential step for their bio-activation, thus offering the explanation for the insignificant cytotoxicity found by the standardized screening models and opening the new horizons for them to be studied more deeply by means of advanced biological models, which would further consider them as pro-drugs.

## 3. Experimental

### 3.1. Materials

K_2_[PtCl_4_], 6-chloropurine-9-riboside, 2,6-dichloropurine-9-riboside, 2-methoxybenzylamine, 3-methoxybenzylamine, 4-methoxybenzylamine, 2,3-dimethoxybenzylamine, 2,5-dimethoxybenzylamine, 2-chlorobenzylamine, 4-chlorobenzylamine, 4-fluorobenzylamine, 4-methylbenzylamine, triethylamine and solvents used were purchased from Sigma-Aldrich Co. (Prague, Czech Republic), Acros Organics Co. (Pardubice, Czech Republic), Lachema Co. (Brno, Czech Republic) and Fluka Co. (Prague, Czech Republic) and used without further purification. 2-hydroxybenzylamine, 3-hydroxybenzylamine, 4-hydroxybenzylamine, 2-hydroxy-3-methoxybenzylamine and 2-hydroxy-5-methylbenzylamine were obtained from the respective methoxy derivatives by the action of the concentrated HBr or HI acids in the presence of acetylacetate.

### 3.2. Methods

Elemental analysis (C, H, N) was performed on a Flash 2000 CHNS Elemental Analyzer (Thermo Scientific, Waltham, MA, USA). Conductivity measurements of 10^–3^ M DMF solutions of the prepared complexes were carried out using a conductometer 340i/SET (WTW) at 25 °C. Simultaneous thermogravimetry (TG) and differential thermal (DTA) analysis was carried out using an Exstar TG/DTA 6200 thermal analyzer (Seiko Instruments Inc., Torrance, CA, USA); dynamic air atmosphere (100 mL min^–1^), 25–900 °C (5.0 °C min^–1^). Infrared spectra (150–600 cm^–1^ region by nujol technique and 400–4,000 cm^–1^ region by ATR technique) were recorded on a Nexus 670 FT-IR (Thermo Nicolet, Waltham, MA, USA). ^1^H, ^13^C and ^195^Pt NMR spectra and 2D correlation experiments (^1^H–^1^H gs-COSY, ^1^H–^1^H gs-ROESY, ^1^H–^13^C gs-HMQC, ^1^H–^13^C gs-HMBC and ^1^H–^15^N gs-HMBC; gs = gradient selected, COSY = correlation spectroscopy, ROESY = rotating frame Overhauser effect spectroscopy, HMQC = heteronuclear multiple quantum coherence and HMBC = heteronuclear multiple bond coherence) of the DMF-d_7_ solutions were measured at 300 K on a Varian 400 device (Varian, Santa Clara, CA, USA) at 400.00 MHz (^1^H), 100.58 MHz (^13^C), 40.53 MHz (^15^N) and 85.78 MHz (^195^Pt). ^1^H and ^13^C spectra were adjusted against the tetramethylsilane (Me_4_Si) signals. ^195^Pt spectra were calibrated against K_2_[PtCl_6_] in D_2_O (0 ppm). ^1^H–^15^N gs-HMBC experiments were obtained at natural abundance and calibrated against the residual signals of the DMF adjusted to 8.03 ppm (^1^H) and 104.7 ppm (^15^N). Splitting of the proton resonances is defined as s = singlet, d = doublet, t = triplet, sx = sextuplet, sep = septuplet, br = broad band, dd = doublet of doublets, tt = triplet of triplets, m = multiplet. ESI− mass spectra (methanol solutions) were obtained by an LCQ Fleet Ion Trap mass spectrometer (Thermo Scientific; QualBrowser software, version 2.0.7, Waltham, MA, USA).

### 3.3. Synthesis

N6-(2-methoxybenzyl)adenosine (**L_1_**), N6-(4-methoxybenzyl)adenosine (**L_2_**), N6-(2-chlorobenzyl)-adenosine (**L_3_**), N6-(4-chlorobenzyl)adenosine (**L_4_**), N6-(2-hydroxybenzyl)adenosine (**L_5_**), N6-(3-hydroxybenzyl)adenosine (**L_6_**), N6-(2-hydroxy-3-methoxybenzyl)adenosine (**L_7_**), N6-(4-fluoro- benzyl)adenosine (**L_8_**), N6-(4-methylbenzyl)adenosine (**L_9_**), 2-chloro-N6-(3-hydroxybenzyl)adenosine (**L_10_**), 2-chloro-N6-(4-hydroxybenzyl)adenosine (**L_11_**), 2-chloro-N6-(2-hydroxy-3-methoxybenzyl)-adenosine (**L_12_**) and 2-chloro-N6-(2-hydroxy-5-methylbenzyl)adenosine (**L_13_**) (see Scheme 1) were synthesized according to the general procedure, which involves the interaction of appropriate purine-based precursor with an excess of amine in an excess of triethylamine [28]. In our case, 1 mmol of 6-chloropurine-9-riboside (for N6-benzyladenosine derivatives **L_1_**–**L_9_**) or 2,6-dichloropurine-9-riboside (for 2-chloro-N6-benzyladenosine derivatives **L_10_**–**L_13_**) reacted with 1.33 mmol of the appropriate benzylamine derivative in *n*-butanol (20 mL) containing triethylamine (1.67 mmol). The reaction mixture was stirred at 90 °C for 4 h and then it was left at -5 °C in the freezer overnight. The white solid was collected by filtration and washed with cold *n*-butanol (2 × 5 mL), cold distilled water (2 × 5 mL) and diethyl ether (2 × 5 mL). In case that not sufficient purity of the sample was achieved (controlled by thin-layer chromatography and elemental analysis), the product was recrystallized from ethanol. The results of elemental analyses, IR and NMR spectroscopy can be found in the Supplementary Material.

The solution 1.0 mmol of **L_1_**–**L_13_** in 50 mL of methanol was slowly poured in the solution of 0.5 mmol of K_2_[PtCl_4_] in a minimum volume of distilled water. The reaction mixture was stirred at room temperature for several days, followed by filtration of the obtained precipitate. The products of the composition *trans-*[PtCl_2_(L_1_)_2_]∙½CH_3_OH (**1**), *trans-*[PtCl_2_(L_2_)_2_]∙CH_3_OH (**2**), *trans-*[PtCl_2_(L_3_)_2_]∙½H_2_O (**3**), *trans-*[PtCl_2_(L_4_)_2_]∙CH_3_OH (**4**), *trans-*[PtCl_2_(L_5_)_2_]∙½CH_3_OH (**5**), *trans-*[PtCl_2_(L_6_)_2_]∙CH_3_OH (**6**), *trans-*[PtCl_2_(L_7_)_2_]∙CH_3_OH (**7**), *trans-*[PtCl_2_(L_8_)_2_]∙½CH_3_OH (**8**), *trans-*[PtCl_2_(L_9_)_2_]∙½CH_3_OH (**9**), *trans-*[PtCl_2_(L_10_)_2_]∙¾CH_3_OH (**10**), *trans-*[PtCl_2_(L_11_)_2_]∙CH_3_OH (**11**), *trans-*[PtCl_2_(L_12_)_2_]∙CH_3_OH (**12**) and *trans-*[PtCl_2_(L_13_)_2_]∙¾CH_3_OH (**13**), generally expressed as *trans-*[PtCl_2_(L_n_)_2_]∙*x*Solv **1**–**13** (Solv = H_2_O or CH_3_OH), were washed by distilled water, methanol and diethyl ether and dried in desiccator over KOH. The physical measurement results are given in the Supplementary Material.

### 3.4. *In Vitro* Cytotoxic Activity Testing

*In vitro* cytotoxicity was studied by an MTT assay [MTT = 3-(4,5-dimethylthiazol-2-yl)-2,5-diphenyltetrazolium bromide] against osteosarcoma (HOS; ECACC No. 87070202) and breast adenocarcinoma (MCF7; ECACC No. 86012803) human cancer cell lines supplied from European Collection of Cell Cultures (ECACC). The cell lines were cultured according to the ECACC instructions, maintained at 37 °C and 5% CO_2_ in a humidified incubator (100% humidity). The studied complexes **1**–**13**, K_2_[PtCl_4_], and *cisplatin* (0.01, 0.1, 1.0, 5.0, 25.0 and 50.0 μM concentration), as well as vehicle (DMF, 0.1%, v/v; positive control to assess the minimal cell damage, *i.e.*, 100% viability) and Triton X-100 (1%, v/v; negative control to assess the maximal cell damage, *i.e.*, 0% viability) were added to the cell suspensions in 96-well culture plates and incubated for 24 h. The MTT assay was measured spectrophotometrically at 540 nm (TECAN, Schoeller Instruments LLC, Prague, Czech Republic). The data from the respective cancer cells were acquired from three independent experiments (conducted in triplicate) using cells from different passages. The resulting IC_50_ values (µM) were calculated from viability curves and the results are presented as arithmetic mean ± SD.

### 3.5. Interactions of the Selected Complexes ***7*** and ***12*** with L-Methionine, Evaluated by Mass Spectrometry

The electrospray-ionization mass spectrometry (ESI-MS) is widely used method for studies of covalent [29] or non-covalent interactions ([30,31] and references therein) of small molecules or ions with biologically relevant molecules. Therefore, we performed the interaction experiments between the two complexes (**7** and **12**), one selected from the group of 2-chloro-substituted derivatives and the second one selected from the 2-unsubstituted group of compounds (dissolved in methanol, at the final concentration of 10 μM) with the physiological concentration of l-Methionine (dissolved in water, at the final concentration of 20 µM), using an LCQ Fleet Ion Trap mass spectrometer, in both the negative and positive ionization modes. The measured solutions were infused directly to the spray-needle by linear pump with the rate of 50 µL/min, and the measured range of ions was set from *m/z* 50 to 1500. No additional tuning was needed to perform the analyses.

## 4. Conclusions

We prepared and thoroughly characterized thirteen platinum(II) dichlorido complexes of the general composition *trans-*[PtCl_2_(L_n_)_2_]∙*x*Solv **1**–**13** (Solv = water or methanol), which represent the first examples of the platinum complexes with N6-benzyladenosine derivatives acting as N-donor ligands. The composition and constitution of the complexes were proved by the multinuclear (^1^H-, ^13^C-, ^195^Pt- and ^15^N-) and two-dimensional NMR spectroscopy. The screening of antitumor activity showed that the complexes **1**–**13** are inactive against the MCF7 and HOS human cancer cell lines (IC_50_ > 50.0 µM). The selected complexes **7** and **12** showed their ability to interact with the physiological levels of sulfur-containing biogenic biomolecule (l-Methionine) by a relatively simple 1:1 exchange mechanism (one L_n_ molecule was replaced by one l-Methionine molecule), thus forming a mixed-nitrogen/sulfur-ligand dichlorido-platinum(II) coordination species, which could be responsible for bio-activation of complexes during the longer time-period. From this point of view, the results of interaction studies support an idea that the studied complexes might be considered as possible pro-drugs. However, to validate such an assumption, the more sophisticated and advanced mechanistic studies have to be undertaken. 

## Supplementary Materials

Supplementary materials can be accessed at: http://www.mdpi.com/1420-3049/18/6/6990/s1.
