# Supplementary material for: N6-Benzyladenosine Derivatives as Novel N-Donor Ligands of Platinum(II) Dichlorido Complexes
Source: Molecules. 2013 Jun 14;18(6):6990–7003. doi: 10.3390/molecules18066990 (PMC6270444; doi:10.3390/molecules18066990)

Supplementary Materials

1. General characterizations of the N6-benzyladenosine derivatives L_1–L_{13} (The results of physical measurements, *i.e.*, elemental analysis, IR and NMR spectroscopy).

L_1: Yield: 90%. Anal. Calcd for $C_{18}H_{21}N_5O_5$: C, 55.8; N, 18.1; H, 5.5. Found: C, 55.5; N, 18.2; H, 5.3%. TLC: one spot. IR, cm^{-1}: 3514m $\nu(O–H)_{aliph}$, 3318m $\nu(N–H)$, 3135m $\nu(C–H)_{ar}$, 2936m $\nu(C–H)_{aliph}$, 1626s $\nu(C=N)_{ar}$, 1245m $(C–O)_{ar}$, 1118m, 1101m, 1055m, 1029m $\nu(C–O)_{aliph}$. ^{1}H-NMR, δ ppm, *J* Hz: 8.42, 1H, *s*, HC^{8}; 8.24, 1H, *s*, HC^{2}; 8.06, 1H, *br*, HN^{6}; 7.29, 1H, *d*, 7.3, HC^{15}; 7.25, 1H, *tt*, 7.7, 1.6, HC^{13}; 7.03, 1H, *d*, 8.2, HC^{12}; 6.89, 1H, *tt*, 7.4, 1.0, HC^{14}; 6.06, 1H, *d*, 6.5, HC^{16}; 5.73, 1H, *m*, $HO^{17'}$; 5.61, 1H, *d*, 6.3, $HO^{20'}$; 5.26, 1H, *d*, 4.0, $HO^{18'}$; 4.86, 2H, *br*, HC^{9}; 4.86, 1H, *q*, 5.3, HC^{17}; 4.36, 1H, *sxt*, 2.6, HC^{18}; 4.14, 1H, *q*, 2.6, HC^{19}; 3.91, 3H, *s*, $HC^{11'}$; 3.83, 1H, *m*, H_aC^{20}; 3.71, 1H, *m*, H_bC^{20}. ^{13}C-NMR, δ ppm: 157.39 (C11), 155.47 (C6), 152.58 (C2), 148.85 (C4), 140.48 (C8), 128.11 (C13), 127.64 (C10), 127.56 (C15), 120.79 (C5), 120.28 (C14), 110.47 (C12), 89.41 (C16), 87.12 (C19), 74.40 (C17), 71.81 (C18), 62.61 (C20), 55.27 (C11′), 38.91 (C9). ^{15}N-NMR, δ ppm, (*J* Hz): $86.41^{8.06,\ (87.8)\ HN6}$ N6; $171.48^{8.42,\ HC8;\ 6.06,\ HC16;\ 4.87,\ HC17}$ N9; $223.89^{8.42,\ HC8;\ 8.24,\ HC2;\ 5.74,\ HO17}$ N3; $232.74^{8.24,\ HC2}$ N1; $240.91^{8.42,\ HC8}$ N7.

L_2: Yield: 93%. Anal. Calcd for $C_{18}H_{21}N_5O_5$: C, 55.8; N, 18.1; H, 5.5. Found: C, 55.3; N, 17.8; H, 5.3%. TLC: one spot. IR, cm^{-1}: 3543m $\nu(O–H)_{aliph}$, 3318m $\nu(N–H)$, 3152m $\nu(C–H)_{ar}$, 2930m $\nu(C–H)_{aliph}$, 1631s $\nu(C=N)_{ar}$, 1250m $(C–O)_{ar}$, 1102m, 1081m, 1056m, 1028m $\nu(C–O)_{aliph}$. ^{1}H-NMR, δ ppm, *J* Hz: 8.41, 1H, *s*, HC^{8}; 8.30, 1H, *br*, HN^{6}; 8.29, 1H, *s*, HC^{2}; 7.40, 2H, *d*, 8.7, $HC^{12,\ 14}$; 6.91, 2H, *d*, 8.7, $HC^{11,\ 15}$; 6.05, 1H, *d*, 6.4, HC^{16}; 5.77, 1H, *br*, $HO^{17'}$; 5.64, 1H, *d*, 5.3, $HO^{20'}$; 5.29, 1H, *br*, $HO^{18'}$; 4.86, 1H, *q*, 5.1, HC^{17}; 4.80, 2H, *br*, HC^{9}; 4.37, 1H, *m*, HC^{18}; 4.15, 1H, *q*, 2.8, HC^{19}; 3.84, 1H, *m*, H_aC^{20}; 3.78, 3H, *s*, $HC^{13'}$; 3.72, 1H, *m*, H_bC^{20}. ^{13}C-NMR, δ ppm: 158.87 (C13), 155.14 (C6), 152.51 (C2), 148.76 (C4), 140.35 (C8), 132.30 (C10), 128.99 (C12, 14), 120.66 (C5), 113.79 (C11, 15), 89.36 (C16), 87.05 (C19), 74.37 (C17), 71.76 (C18), 62.55 (C20), 54.97 (C13′), 42.91 (C9). ^{15}N-NMR, δ ppm, (*J* Hz): $92.43^{8.30,\ (86.4)\ HN6}$ N6; $171.75^{8.41,\ HC8;\ 6.06,\ (5.5)\ HC16}$ N9; $223.22^{8.29,\ (12.0)\ HC2}$ N3; $232.01^{8.30,\ (13.4)\ HN6}$ N1; $240.58^{8.41,\ (9.8)\ HC8}$ N7.

L_3: Yield: 92%. Anal. Calcd for $C_{17}H_{18}N_5O_4Cl$: C, 52.1; N, 17.9; H, 4.6. Found: C, 52.0; N, 17.6; H, 4.7%. TLC: one spot. IR, cm^{-1}: 3545m $\nu(O–H)_{aliph}$, 3307m $\nu(N–H)$, 3144m $\nu(C–H)_{ar}$, 2933m $\nu(C–H)_{aliph}$, 1618s $\nu(C=N)_{ar}$, 1179w $(C–Cl)_{ar}$, 1116m, 1095m, 1052m, 1029m $\nu(C–O)_{aliph}$. ^{1}H-NMR, δ ppm, *J* Hz: 8.47, 1H, *s*, HC^{8}; 8.38, 1H, *br*, HN^{6}; 8.27, 1H, *s*, HC^{2}; 7.47, 2H, *mm*, $HC^{12,\ 15}$; 7.31, 2H, *mm*, $HC^{13,\ 14}$; 6.08, 1H, *d*, 6.5, HC^{16}; 5.72, 1H, *q*, 3.7, $HO^{17'}$; 5.65, 1H, *d*, 6.0, $HO^{20'}$; 5.31, 1H, *d*, 3.7, $HO^{18'}$; 4.96, 2H, *br*, HC^{9}; 4.87, 1H, *q*, 5.5, HC^{17}; 4.39, 1H, *m*, HC^{18}; 4.16, 1H, *m*, HC^{19}; 3.84, 1H, *m*, H_aC^{20}; 3.73, 1H, *m*, H_bC^{20}. ^{13}C-NMR, δ ppm: 155.20 (C6), 152.53 (C2), 148.98 (C4), 140.63 (C8), 137.21 (C10), 132.48 (C11), 129.37 (C15), 128.60 (C12), 128.57 (C13), 127.23 (C14), 120.74 (C5), 89.32 (C16), 87.04 (C19), 74.43 (C17), 71.74 (C18), 62.53 (C20), 41.54 (C9). ^{15}N-NMR, δ ppm, (*J* Hz): $85.45^{8.38,\ (83.2)\ HN6}$ N6; $172.41^{8.47,\ (6.8)\ HC8;\ 6.08,\ (6.5)\ HC16;\ 4.87,\ (7.4)\ HC17}$ N9; $225.02^{8.27,\ (15.1)\ HC2}$ N3; $233.06^{8.27,\ (15.1)\ HC2}$ N1; $241.10^{8.47,\ (10.2)\ HC8}$ N7.

L_4: Yield: 90%. Anal. Calcd for $C_{17}H_{18}N_5O_4Cl$: C, 52.1; N, 17.9; H, 4.6. Found: C, 51.8; N, 17.1; H, 4.5%. TLC: one spot. IR, cm^{-1}: 3544m $\nu(O–H)_{aliph}$, 3337m $\nu(N–H)$, 3147m $\nu(C–H)_{ar}$, 2926m $\nu(C–$

H)$_{aliph}$, 1625s ν(C=N)$_{ar}$, 1155w (C–Cl)$_{ar}$, 1119m, 1090m, 1051m, 1017w ν(C–O)$_{aliph}$. ^{1}H-NMR, *δ* ppm, *J* Hz: 8.43, 1H, *s*, HC^{8}; 8.42, 1H, *br*, HN^{6}; 8.27, 1H, *s*, HC^{2}; 7.49, 2H, *d*, 8.5, HC$^{12,\ 14}$; 7.39, 2H, *d*, 8.5, HC$^{11,\ 15}$; 6.05, 1H, *d*, 6.4, HC^{16}; 5.71, 1H, *br*, HO$^{17'}$; 5.60, 1H, *d*, 6.1, HO$^{20'}$; 5.26, 1H, *d*, 4.2, HO$^{18'}$; 4.85, 1H, *q*, 6.2, HC^{17}; 4.85, 2H, *br*, HC^{9}; 4.36, 1H, *m*, HC^{18}; 4.14, 1H, *q*, 2.7, HC^{19}; 3.83, 1H, *m*, H$_{a}$C^{20}; 3.71, 1H, *m*, H$_{b}$C^{20}. ^{13}C-NMR, *δ* ppm: 155.17 (C6), 152.55 (C2), 148.92 (C4), 140.52 (C8), 139.59 (C10), 131.93 (C13), 129.50 (C12, 14), 128.46 (C11, 15), 120.73 (C5), 89.35 (C16), 87.09 (C19), 74.42 (C17), 71.78 (C18), 62.58 (C20), 42.91 (C9). ^{15}N-NMR, *δ* ppm, (*J* Hz): $89.51^{8.42,\ (86.4)\ HN6}$ N6; $171.61^{8.43,\ (6.9)\ HC8;\ 6.06,\ (6.3)\ HC16;\ 4.85,\ HC17}$ N9; $223.91^{8.43,\ (10.2)\ HC8;\ 8.27,\ (13.0)\ HC2}$ N3; $232.46^{8.27,\ (14.4)\ HC2}$ N1; $241.11^{8.43,\ (10.9)\ HC8}$ N7.

L$_{5}$: Yield: 91%. Anal. Calcd for $C_{17}H_{19}N_5O_5$: C, 54.7; N, 18.8; H, 5.1. Found: C, 54.5; N, 19.0; H, 4.7%. TLC: one spot. IR, cm^{-1}: 3411m ν(O–H)$_{aliph}$, 3295m ν(N–H), 3153m ν(C–H)$_{ar}$, 2926m ν(C–H)$_{aliph}$, 1639s, 1625s ν(C=N)$_{ar}$, 1234m (C–O)$_{ar}$, 1130m, 1101m, 1056m, 1040m ν(C–O)$_{aliph}$. ^{1}H-NMR, *δ* ppm, *J* Hz: 10.40, 1H, br, HO$^{11'}$; 8.45, 1H, *s*, HC^{8}; 8.33, 1H, *s*, HC^{2}; 8.31, 1H, *br*, HN^{6}; 7.32, 1H, *dd*, 7.7, 1.2, HC^{15}; 7.13, 1H, *tt*, 7.7, 1.2, HC^{13}; 6.92, 1H, *d*, 8.0, HC^{12}; 6.79, 1H, *t*, 7.5, HC^{14}; 6.08, 1H, *d*, 6.2, HC^{16}; 5.70, 1H, *br*, HO$^{17'}$; 5.68, 1H, *br*, HO$^{20'}$; 5.30, 1H, *br*, HO$^{18'}$; 4.85, 1H, *t*, 5.8, HC^{17}; 4.81, 2H, *br*, HC^{9}; 4.39, 1H, *m*, HC^{18}; 4.16, 1H, *q*, 2.9, HC^{19}; 3.85, 1H, *m*, H$_{a}$C^{20}; 3.72, 1H, *m*, H$_{b}$C^{20}. ^{13}C-NMR, *δ* ppm: 155.84 (C11), 154.87 (C6), 152.20 (C2), 148.68 (C4), 140.54 (C8), 129.46 (C15), 128.44 (C13), 125.98 (C10), 120.57 (C5), 119.27 (C14), 116.11 (C12), 89.27 (C16), 86.96 (C19), 74.44 (C17), 71.66 (C18), 62.45 (C20), 39.66 (C9). ^{15}N-NMR, *δ* ppm, (*J* Hz): $90.54^{8.31,\ (89.5)\ HN6}$ N6; $172.09^{8.45,\ (6.9)\ HC8;\ 6.08,\ HC16}$ N9; $223.64^{8.45,\ HC8;\ 8.33,\ (13.0)\ HC2}$ N3; $227.09^{8.33,\ (13.0)\ HC2}$ N1; $240.64^{8.45,\ (11.6)\ HC8}$ N7.

L$_{6}$: Yield: 90%. Anal. Calcd for $C_{17}H_{19}N_5O_5$: C, 54.7; N, 18.8; H, 5.1. Found: C, 54.4; N, 18.5; H, 4.9%. TLC: one spot. IR, cm^{-1}: 3481w, 3430m ν(O–H)$_{aliph}$, 3316m ν(N–H), 3157m ν(C–H)$_{ar}$, 2929m ν(C–H)$_{aliph}$, 1624s ν(C=N)$_{ar}$, 1216m (C–O)$_{ar}$, 1126m, 1098m, 1078m, 1054m ν(C–O)$_{aliph}$. ^{1}H-NMR, *δ* ppm, *J* Hz: 9.48, 1H, br, HO$^{12'}$; 8.42, 1H, *s*, HC^{8}; 8.29, 1H, *br*, HN^{6}; 8.27, 1H, *s*, HC^{2}; 7.14, 1H, *t*, 7.9, HC^{14}; 6.95, 1H, *t*, 2.0, HC^{11}; 6.88, 1H, *d*, 7.5, HC^{15}; 6.72, 1H, *dd*, 8.1, 2.7, HC^{13}; 6.06, 1H, *d*, 6.6, HC^{16}; 5.77, 1H, *q*, 3.6, HO$^{17'}$; 5.63, 1H, *d*, 6.3, HO$^{20'}$; 5.28, 1H, *d*, 4.1, HO$^{18'}$; 4.86, 1H, *q*, 5.8, HC^{17}; 4.82, 2H, *br*, HC^{9}; 4.37, 1H, *q*, 3.8, HC^{18}; 4.15, 1H, *q*, 2.8, HC^{19}; 3.83, 1H, *m*, H$_{a}$C^{20}; 3.72, 1H, *m*, H$_{b}$C^{20}. ^{13}C-NMR, *δ* ppm: 158.13 (C12), 155.22 (C6), 152.52 (C2), 148.78 (C4), 141.92 (C10), 140.40 (C8), 129.34 (C14), 120.66 (C5), 118.15 (C15), 114.40 (C11), 113.85 (C13), 89.37 (C16), 87.07 (C19), 74.35 (C17), 71.78 (C18), 62.57 (C20), 43.35 (C9). ^{15}N-NMR, *δ* ppm, (*J* Hz): $89.91^{8.29,\ (88.8)\ HN6}$ N6; $170.89^{8.42,\ (7.7)\ HC8;\ 6.06,\ (6.4)\ HC16;\ 4.87,\ HC17}$ N9; $223.47^{8.42,\ HC8;\ 8.27,\ (10.5)\ HC2}$ N3; $232.36^{8.29,\ HN6}$ N1; $240.51^{8.42,\ (11.8)\ HC8}$ N7.

L$_{7}$: Yield: 85%. Anal. Calcd for $C_{18}H_{21}N_5O_6$: C, 53.6; N, 17.4; H, 5.3. Found: C, 53.3; N, 17.3; H, 5.4%. TLC: one spot. IR, cm^{-1}: 3491m ν(O–H)$_{aliph}$, 3327m ν(N–H), 3133m ν(C–H)$_{ar}$, 2928m ν(C–H)$_{aliph}$, 1633s ν(C=N)$_{ar}$, 1234m (C–O)$_{ar}$, 1119m, 1080m, 1067m ν(C–O)$_{aliph}$. ^{1}H-NMR, *δ* ppm, *J* Hz: 9.64, 1H, *br*, HO$^{11'}$; 8.44, 1H, *s*, HC^{8}; 8.31, 1H, *s*, HC^{2}; 8.22, 1H, *br*, HN^{6}; 6.92, 1H, *d*, 8.2, HC^{15}; 6.90, 1H, *d*, 8.2, HC^{13}; 6.74, 1H, *t*, 8.2, HC^{14}; 6.06, 1H, *d*, 6.2, HC^{16}; 5.67, 1H, *qui*, 4.3, HO$^{17'}$; 5.64, 1H, *d*, 5.5, HO$^{20'}$; 5.26, 1H, *d*, 3.1, HO$^{18'}$; 4.84, 1H, *qui*, 5.4, HC^{17}; 4.83, 2H, *br*, HC^{9}; 4.36, 1H, *m*, HC^{18}; 4.14, 1H, *q*, 2.9, HC^{19}; 3.85, 1H, *m*, H$_{a}$C^{20}; 3.83, 3H, *s*, HC$^{12'}$; 3.72, 1H, *m*, H$_{b}$C^{20}. ^{13}C-NMR, *δ*

ppm: 154.98 (C6), 152.31 (C2), 148.74 (C4), 148.39 (C12), 144.97 (C11), 140.56 (C8), 126.46 (C10), 121.07 (C15), 120.65 (C5), 118.96 (C14), 111.05 (C13), 89.32 (C16), 87.02 (C19), 74.46 (C17), 71.72 (C18), 62.51 (C20), 55.79 (C12′), 39.42 (C9). ^{15}N-NMR, *δ* ppm, (*J* Hz): 90.59$^{8.22,\ (94.1)\ \mathrm{HN6}}$ N6; 172.13$^{8.44,\ (7.2)\ \mathrm{HC8};\ 6.06,\ (6.7)\ \mathrm{HC16}}$ N9; 223.62$^{8.31,\ (12.7)\ \mathrm{HC2}}$ N3; 227.75$^{8.31,\ (12.7)\ \mathrm{HC2}}$ N1; 241.18$^{8.44,\ (11.5)\ \mathrm{HC8}}$ N7.

L$_8$: Yield: 92%. Anal. Calcd for $C_{17}H_{18}N_5O_4F$: C, 54.4; N, 18.7; H, 4.8. Found: C, 54.3; N, 18.4; H, 4.8%. TLC: one spot. IR, cm^{-1}: 3568m ν(O–H)$_{aliph}$, 3324m ν(N–H), 3141m ν(C–H)$_{ar}$, 2924m ν(C–H)$_{aliph}$, 1623s ν(C=N)$_{ar}$, 1220m (C–F)$_{ar}$, 1119m, 1100m, 1049m, 1029m ν(C–O)$_{aliph}$. ^{1}H-NMR, *δ* ppm, *J* Hz: 8.43, 1H, *s*, HC^8; 8.39, 1H, *br*, HN^6; 8.28, 1H, *s*, HC^2; 7.52, 2H, *m*, HC$^{11,\ 15}$; 7.15, 2H, *tt*, 8.8, 2.3, HC$^{12,\ 14}$; 6.06, 1H, *d*, 6.4, HC^{16}; 5.73, 1H, *q*, 3.9, HO$^{17′}$; 5.62, 1H, *d*, 6.3, HO$^{20′}$; 5.27, 1H, *d*, 4.3, HO$^{18′}$; 4.86, 1H, *q*, 5.8, HC^{17}; 4.86, 2H, *d*, 5.6, HC^9; 4.37, 1H, *sxt*, 2.4, HC^{18}; 4.15, 1H, *q*, 2.7, HC^{19}; 3.83, 1H, *m*, H$_a$C^{20}; 3.72, 1H, *m*, H$_b$C^{20}. ^{13}C-NMR, *δ* ppm: 163.07, 160.66 (C13), 155.13 (C6), 152.54 (C2), 148.89 (C4), 140.49 (C8), 136.66 (C10), 129.70, 129.62 (C11, 15), 120.74 (C5), 115.17, 114.96 (C12, 14), 89.37 (C16), 87.08 (C19), 74.40 (C17), 71.78 (C18), 62.58 (C20), 42.82 (C9). ^{15}N-NMR, *δ* ppm, (*J* Hz): 90.67$^{8.39,\ (90.5)\ \mathrm{HN6}}$ N6; 171.66$^{8.43,\ (5.9)\ \mathrm{HC8}}$ N9; 224.78$^{8.28,\ (12.8)\ \mathrm{HC2}}$ N3; 232.55$^{8.28,\ (12.8)\ \mathrm{HC2}}$ N1; 240.98$^{8.43,\ (10.8)\ \mathrm{HC8}}$ N7.

L$_9$: Yield: 92%. Anal. Calcd for $C_{18}H_{21}N_5O_4$: C, 58.2; N, 18.9; H, 5.7. Found: C, 58.4; N, 18.5; H, 5.8%. TLC: one spot. IR, cm^{-1}: 3519m ν(O–H)$_{aliph}$, 3327m ν(N–H), 3156m ν(C–H)$_{ar}$, 2922m ν(C–H)$_{aliph}$, 1628s ν(C=N)$_{ar}$, 1121m, 1095m, 1080m, 1057m ν(C–O)$_{aliph}$. ^{1}H-NMR, *δ* ppm, *J* Hz: 8.41, 1H, *s*, HC^8; 8.31, 1H, *br*, HN^6; 8.28, 1H, *s*, HC^2; 7.34, 2H, *d*, 8.0, HC^{11,15}; 7.14, 2H, *d*, 7.8, HC^{12,14}; 6.06, 1H, *d*, 6.5, HC^{16}; 5.76, 1H, *q*, 3.9, HO$^{17′}$; 5.63, 1H, *d*, 6.4, HO$^{20′}$; 5.28, 1H, *d*, 4.4, HO$^{18′}$; 4.86, 1H, *q*, 5.9, HC^{17}; 4.83, 2H, *br*, HC^9; 4.37, 1H, *sxt*, 2.6, HC^{18}; 4.15, 1H, *q*, 2.8, HC^{19}; 3.83, 1H, *m*, H$_a$C^{20}; 3.72, 1H, *m*, H$_b$C^{20}; 2.28, 3H, *s*, HC$^{13′}$. ^{13}C-NMR, *d$_7$*-DMFA, TMS, *δ* (ppm), 25°C: 155.23 (C6), 152.54 (C2), 148.78 (C4), 140.40 (C8), 137.38 (C10), 136.27 (C13), 129.02 (C12,14), 127.63 (C11,15), 120.69 (C5), 89.39 (C16), 87.09 (C19), 74.38 (C17), 71.79 (C18), 62.59 (C20), 43.24 (C9); 20.43 (C13′). ^{15}N-NMR, *δ* ppm, (*J* Hz): 92.30$^{8.31,\ (90.1)\ \mathrm{HN6}}$ (N6); 171.35$^{8.41,\ \mathrm{HC8};\ 6.06,\ (6.0)\ \mathrm{HC16};\ 4.86,\ \mathrm{HC17}}$ (N9); 223.46$^{8.28,\ (11.2)\ \mathrm{HC2};\ 5.77,\ \mathrm{HO17′}}$ (N3); 232.28$^{8.28,\ (13.3)\ \mathrm{HC2}}$ (N1); 241.06$^{8.41,\ (11.3)\ \mathrm{HC8}}$ (N7).

L$_{10}$: Yield: 90%. Anal. Calcd for $C_{17}H_{18}N_5O_5Cl$: C, 50.1; N, 17.2; H, 4.5. Found: C, 50.3; N, 17.4; H, 4.3%. TLC: one spot. IR, cm^{-1}: 3303m ν(N–H), 3112m ν(C–H)$_{ar}$, 2932m ν(C–H)$_{aliph}$, 1616s ν(C=N)$_{ar}$, 1219m (C–O)$_{ar}$, 1099m, 1075m, 1046m ν(C–O)$_{aliph}$. ^{1}H-NMR, *δ* ppm, *J* Hz: 9.53, 1H, *br*, HO$^{12′}$; 8.77, 1H, *t*, 6.2, HN^6; 8.47, 1H, *s*, HC^8; 7.16, 1H, *t*, 7.9, HC^{14}; 6.94, 1H, *s*, HC^{11}; 6.88, 1H, *d*, 7.6, HC^{15}; 6.74, 1H, *dd*, 8.3, 1.6, HC^{13}; 6.02, 1H, *d*, 6.0, HC^{16}; 5.68, 1H, *br*, HO$^{17′}$; 5.27, 2H, *br*, HO$^{18′,20′}$; 4.76, 1H, *m*, HC^{17}; 4.76, 2H, *d*, 6.2, HC^9; 4.37, 1H, *t*, 3.6, HC^{18}; 4.13, 1H, *q*, 3.3, HC^{19}; 3.84, 1H, *m*, H$_a$C^{20}; 3.75, 1H, *m*, H$_b$C^{20}. ^{13}C-NMR, *δ* ppm: 158.14 (C12), 155.58 (C6), 153.78 (C2), 150.10 (C4), 141.06 (C10), 140.43 (C8), 129.41 (C14), 119.28 (C5), 118.22 (C15), 114.44 (C11), 114.02 (C13), 88.60 (C16), 86.69 (C19), 74.63 (C17), 71.38 (C18), 62.20 (C20), 43.55 (C9). ^{15}N-NMR, *δ* ppm, (*J* Hz): 94.78$^{8.77,\ (94.4)\ \mathrm{HN6}}$ N6; 172.12$^{8.47,\ (8.0)\ \mathrm{HC8};\ 6.06,\ (4.8)\ \mathrm{HC16};\ 4.76,\ \mathrm{HC17}}$ N9; 221.18$^{8.47,\ \mathrm{HC8}}$ N3; 227.74$^{8.77,\ \mathrm{HN6}}$ N1; 241.18$^{8.47,\ (11.2)\ \mathrm{HC8}}$ N7.

L$_{11}$: Yield: 92%. Anal. Calcd for $C_{17}H_{18}N_5O_5Cl$: C, 50.1; N, 17.2; H, 4.5. Found: C, 50.1; N, 17.3; H, 4.2%. TLC: one spot. IR, cm^{-1}: 3268s ν(N–H), 3122s ν(C–H)$_{ar}$, 2934s ν(C–H)$_{aliph}$, 1650s, 1613s

ν(C=N)$_{ar}$, 1220m (C–O)$_{ar}$, 1175m (C–Cl)$_{ar}$, 1118m, 1064m, 1049m ν(C–O)$_{aliph}$. ^{1}H-NMR, *δ* ppm, *J* Hz: 9.50, 1H, *br*, HO$^{13'}$; 8.68, 1H, *t*, 6.2, HN^{6}; 8.44, 1H, *s*, HC^{8}; 7.30, 2H, *dd*, 8.6, 2.2, HC^{11,15}; 6.82, 2H, *dd*, 8.2, 2.2, HC^{12,14}; 6.01, 1H, *d*, 6.5, HC^{16}; 5.68, 1H, *d*, 5.9, HO$^{17'}$; 5.29, 2H, *mm*, HO$^{18',20'}$; 4.76, 1H, *sxt*, 5.7, HC^{17}; 4.71, 2H, *d*, 6.0, HC^{9}; 4.37, 1H, *q*, 3.8, HC^{18}; 4.13, 1H, *q*, 3.2, HC^{19}; 3.84, 1H, *m*, H$_{a}$C^{20}; 3.75, 1H, *m*, H$_{b}$C^{20}. ^{13}C-NMR, *δ* ppm: 157.12 (C13), 155.41 (C6), 153.74 (C2), 149.99 (C4), 140.32 (C8), 129.78 (C10), 129.18 (C11, 15), 119.25 (C5), 115.21 (C12, 14), 88.60 (C16), 86.65 (C19), 74.60 (C17), 71.35 (C18), 62.18 (C20), 43.30 (C9). ^{15}N-NMR, *δ* ppm, (*J* Hz): 97.99$^{8.68,\ (93.9)\ HN6}$ N6; 172.72$^{8.44,\ (6.1)\ HC8;\ 6.01,\ HC16;\ 4.76,\ (3.5)\ HC17}$ N9; 221.45$^{8.44,\ HC8}$ N3; 227.95$^{8.68,\ HN6}$ N1; 241.59$^{8.44,\ (11.4)\ HC8}$ N7.

L$_{12}$: Yield: 89%. Anal. Calcd for $C_{18}H_{20}N_5O_6Cl$: C, 49.4; N, 16.0; H, 4.6. Found: C, 49.6; N, 15.9; H, 4.3%. TLC: one spot. IR, cm^{-1}: 3532m, 3453m ν(O–H)$_{aliph}$, 3333s ν(N–H), 3112m ν(C–H)$_{ar}$, 2941m ν(C–H)$_{aliph}$, 1642s ν(C=N)$_{ar}$, 1211m (C–O)$_{ar}$, 1159w (C–Cl)$_{ar}$, 1123m, 1076m, 1056s, 1011m ν(C–O)$_{aliph}$. ^{1}H-NMR, *δ* ppm, *J* Hz: 9.06, 1H, *br*, HO$^{11'}$; 8.50, 1H, *t*, 6.0, HN^{6}; 8.48, 1H, *s*, HC^{8}; 6.91, 1H, *d*, 8.0, HC^{15}; 6.89, 1H, *d*, 8.0, HC^{13}; 6.75, 1H, *t*, 8.0, HC^{14}; 6.01, 1H, *d*, 6.0, HC^{16}; 5.68, 1H, *br*, HO$^{17'}$; 5.31, 1H, *br*, HO$^{20'}$; 5.27, 1H, *br*, HO$^{18'}$; 4.82, 2H, *d*, 6.0, HC^{9}; 4.75, 1H, *t*, 5.6, HC^{17}; 4.36, 1H, *t*, 4.2, HC^{18}; 4.12, 1H, *q*, 3.7, HC^{19}; 3.84, 3H, *s*, HC$^{12'}$; 3.83, 1H, *m*, H$_{a}$C^{20}; 3.74, 1H, *m*, H$_{b}$C^{20}. ^{13}C-NMR, *δ* ppm: 155.73 (C6), 153.75 (C2), 150.06 (C4), 147.94 (C12), 144.64 (C11), 140.52 (C8), 125.58 (C10), 120.39 (C15), 119.40 (C5), 118.89 (C14), 110.82 (C13), 88.66 (C16), 86.72 (C19), 74.73 (C17), 71.42 (C18), 62.23 (C20), 55.82 (C12′), 39.37 (C9). ^{15}N-NMR, *δ* ppm, (*J* Hz): 93.46$^{8.50,\ (94.0)\ HN6;\ 4.82,\ HC9}$ N6; 172.53$^{8.48,\ (7.0)\ HC8;\ 6.01,\ (5.7)\ HC16}$ N9; 221.44$^{8.48,\ (11,3)\ HC8}$ N3; 227.07$^{8.50,\ HN6}$ N1; 241.80$^{8.48,\ (13.0)\ HC8}$ N7.

L$_{13}$: Yield: 86%. Anal. Calcd for $C_{18}H_{20}N_5O_5Cl$: C, 51.3; N, 16.6; H, 4.8. Found: C, 51.5; N, 16.3; H, 4.3%. TLC: one spot. IR, cm^{-1}: 3293s ν(N–H), 3119m ν(C–H)$_{ar}$, 2924m ν(C–H)$_{aliph}$, 1617s ν(C=N)$_{ar}$, 1221m (C–O)$_{ar}$, 1163w (C–Cl)$_{ar}$, 1117m, 1076m, 1049m ν(C–O)$_{aliph}$. ^{1}H-NMR, *δ* ppm, *J* Hz: 9.65, 1H, *s*, HO$^{11'}$; 8.51, 1H, *t*, 6.0, HN^{6}; 8.48, 1H, *s*, HC^{8}; 7.12, 1H, *s*, HC^{15}; 6.94, 1H, *d*, 8.1, HC^{12}; 6.83, 1H, *d*, 8.1, HC^{13}; 6.02, 1H, *d*, 5.9, HC^{16}; 5.71, 1H, *d*, 5.6, HO$^{17'}$; 5.32, 1H, *d*, 4.3, HO$^{18'}$; 5.29, 1H, *t*, 5.3, HO$^{20'}$; 4.78, 2H, *d*, 5.7, HC^{9}; 4.76, 1H, *q*, 5.1, HC^{17}; 4.37, 1H, *q*, 3.9, HC^{18}; 4.14, 1H, *q*, 3.1, HC^{19}; 3.84, 1H, *m*, H$_{a}$C^{20}; 3.75, 1H, *m*, H$_{b}$C^{20}; 2.19, 3H, *s*, HC$^{14'}$. ^{13}C-NMR, *δ* ppm: 155.53 (C6), 153.62 (C2), 153.32 (C11), 149.91 (C4), 140.45 (C8), 129.29 (C15), 128.67 (C12), 127.88 (C10), 124.80 (C14), 119.30 (C5), 115.37 (C13), 88.61 (C16), 86.64 (C19), 74.65 (C17), 71.32 (C18), 62.13 (C20), 39.57 (C9), 19.93 (C14′). ^{15}N-NMR, *δ* ppm, (*J* Hz): 93.99$^{8.51,\ (93.9)\ HN6;\ 4.78,\ HC9}$ N6; 172.31$^{8.48,\ HC8;\ 6.02,\ (5.0)\ HC16;\ 4.76,\ HC17}$ N9; 221.15$^{8.48,\ HC8}$ N3; 226.04$^{8.51,\ HN6}$ N1; 241.45$^{8.48,\ (9.1)\ HC8}$ N7.

2. General characterizations of the complexes 1–13 (The results of physical measurements, *i.e.* elemental and thermal analyses, ESI– mass spectrometry, IR and NMR spectroscopy)

1: Yield: 70%. Anal. Calcd for $C_{36}H_{42}N_{10}Cl_2O_{10}Pt \cdot ½CH_3OH$: C, 41.5; N, 13.3; H, 4.2. Found: C, 41.3; N, 13.2; H, 4.1%. IR, cm^{-1}: 3499m ν(O–H)$_{aliph}$, 3354m ν(N–H), 3129m ν(C–H)$_{ar}$, 2936m ν(C–H)$_{aliph}$, 1615s ν(C=N)$_{ar}$, 1248m (C-O)$_{ar}$, 1082m, 1055m ν(C–O)$_{aliph}$, 530s ν(Pt–N), 345m ν(Pt–Cl). ^{1}H-NMR, *δ* ppm, *J* Hz, ($\Delta\delta = \delta_{comple\ x} - \delta_{ligand}$): 9.26, 1H, *s*, HC^{8} (0.84); 8.88, 1H, *t*, 6.2, HN^{6} (0.82); 8.15, 1H, *s*, HC^{2} (-0.09); 7.49, 1H, *d*, 7.3, HC^{15} (0.20); 7.26, 1H, *t*, 7.7, HC^{13} (0.01); 7.01, 1H, *d*, 8.2, HC^{12} (-0.02); 6.84, 1H, *t*, 7.4, HC^{14} (-0.05); 6.20, 1H, *d*, 5.7, HC^{16} (0.14); 5.83, 1H, *d*, 5.8, HO$^{17'}$ (0.10); 5.53, 1H, *m*,

$HO^{20'}$ (-0.08); 5.35, 1H, *d*, 4.6, $HO^{18'}$ (0.09); 4.93, 2H, *d*, 6.1, HC^{9} (0.07); 4.82, 1H, *q*, 5.3, HC^{17} (-0.04); 4.39, 1H, *q*, 4.0, HC^{18} (0.03); 4.19, 1H, *q*, 3.2, HC^{19} (0.05); 3.90, 1H, *m*, H_aC^{20} (0.07); 3.80, 3H, *s*, $HC^{11'}$ (-0.11); 3.78, 1H, *m*, H_bC^{20} (0.07). ^{13}C-NMR, δ ppm, ($\Delta\delta$): 157.62 (C11, 0.23); 153.93 (C2, 1.35); 153.11 (C6, -2.36); 148.03 (C4, -0.82); 143.27 (C8, 2.79); 128.63 (C13, 0.52); 128.18 (C15, 0.62); 126.39 (C10, -1.25); 120.23 (C14, -0.05); 116.72 (C5, -4.07); 110.52 (C12, 0.05); 90.08 (C16, 0.67); 87.26 (C19, 0.14); 74.98 (C17, 0.58); 71.16 (C18, -0.65); 61.97 (C20, -0.64); 55.27 (C11′, 0.00); 40.06 (C9, 1.15). ^{15}N-NMR, δ ppm, (*J* Hz), ($\Delta\delta$): $94.88^{8.88,\ (92.3)\ HN6}$ N6, (8.47); $135.88^{9.26,\ HC8}$ N7, (-105.03); $177.08^{9.26,\ HC8;\ 6.20,\ HC16;\ 4.82,\ HC17}$ N9, (5.60); $225.06^{8.15,\ HC2}$ N3, (1.17); $236.56^{8.88,\ HN6}$ N1, (3.82). ^{195}Pt-NMR, δ ppm: -2077.10. ESI– MS (methanol, *m/z*): 1074.2 (calc. 1074.2; 100%) $[PtCl_3(L_1)_2]^-$, 1038.2 (calc. 1038.2; 10%) $[PtCl_2(L_1)_2\text{-}H]^-$, 906.2 (calc. 906.2; 60%) $[PtCl_2(L_1)(L_1`)\text{-}H]^-$, 870.4 (calc. 870.2; 10%) $[PtCl(L_1)(L_1`)\text{-}2H]^-$, 483.1 (calc. 483.0; 5%) $[PtCl(L_1`)\text{-}2H]^-$. TG/DTA data: weight loss of 1.4% found between 31–100 °C (1.5% calcd. for $0.5CH_3OH$); decomposition began at 100 °C and finished at 595 °C with a weight loss of 78.6%; exothermic peaks at 324 and 470 °C; total weight loss of 80.0% (calc. to PtO residue: 80.1%).

2: Yield: 75%. Anal. Calcd for $C_{36}H_{42}N_{10}Cl_2O_{10}Pt{\cdot}CH_3OH$: C, 41.4; N, 13.1; H, 4.3. Found: C, 41.4; N, 13.5; H, 4.1%. IR, cm^{-1}: 3359m ν(N–H), 3129m $\nu(C\text{–}H)_{ar}$, 2946m $\nu(C\text{–}H)_{aliph}$, 1611s $\nu(C{=}N)_{ar}$, 1244m $(C\text{-}O)_{ar}$, 1082m, 1056m $\nu(C\text{–}O)_{aliph}$, 511s ν(Pt–N), 339m ν(Pt–Cl). ^{1}H-NMR, δ ppm, *J* Hz, ($\Delta\delta$): 9.25, 1H, *s*, HC^{8} (0.84); 8.82, 1H, *t*, 6.0, HN^{6} (0.52); 8.47, 1H, *s*, HC^{2} (0.18); 7.48, 2H, *d*, 8.0, $HC^{12,14}$ (0.08); 6.86, 2H, *d*, 8.1, $HC^{11,15}$ (-0.05); 6.20, 1H, *d*, 5.2, HC^{16} (0.15); 5.85, 1H, *d*, 5.7, $HO^{17'}$ (0.08); 5.58, 1H, *t*, 5.6, $HO^{20'}$ (-0.06); 5.39, 1H, *d*, 4.3, $HO^{18'}$ (0.10); 4.85, 2H, *d*, 5.8, HC^{9} (0.06); 4.83, 1H, *q*, 5.2, HC^{17} (-0.03); 4.41, 1H, *q*, 4.0, HC^{18} (0.04); 4.20, 1H, *q*, 2.9, HC^{19} (0.05); 3.92, 1H, *m*, H_aC^{20} (0.08); 3.78, 1H, *m*, H_bC^{20} (0.06); 3.77, 3H, *s*, $HC^{13'}$ (-0.01). ^{13}C-NMR, δ ppm, ($\Delta\delta$): 159.07 (C13, 0.20); 153.92 (C2, 1.41); 152.86 (C6, -2.28); 147.91 (C4, -0.85); 143.27 (C8, 2.92); 130.65 (C10, -1.65); 129.02 (C12, 14, 0.03); 116.63 (C5, -4.03); 113.88 (C11, 15, 0.09); 90.14 (C16, 0.78); 87.18 (C19, 0.13); 75.00 (C17, 0.63); 71.06 (C18, -0.70); 61.91 (C20, -0.64); 55.01 (C13′, 0.04); 44.11 (C9, 1.20). ^{15}N-NMR, δ ppm, (*J* Hz), ($\Delta\delta$): $99.10^{8.82,\ (91.3)\ HN6}$ N6, (6.67); $135.56^{9.25,\ HC8}$ N7, (-105.02); $177.92^{9.25,\ HC8;\ 6.20,\ HC16;\ 4.83,\ HC17}$ N9, (6.17); $225.22^{9.25,\ HC8;\ 8.47,\ (15.3)\ HC2}$ N3, (2.00); $237.04^{8.82,\ HN6}$ N1, (5.03). ^{195}Pt-NMR, δ (ppm): -2072.08. ESI– MS (methanol, *m/z*): 1073.9 (calc. 1074.2; 100%) $[PtCl_3(L_2)_2]^-$, 1037.8 (calc. 1038.2; 45%) $[PtCl_2(L_2)_2\text{-}H]^-$, 905.8 (calc. 906.2; 70%) $[PtCl_2(L_2)(L_2`)\text{-}H]^-$, 870.0 (calc. 870.2; 30%) $[PtCl(L_2)(L_2`)\text{-}2H]^-$, 483.0 (calc. 483.0; 5%) $[PtCl(L_2`)\text{-}2H]^-$. TG/DTA data: weight loss of 2.0% found between 30–149 °C (2.2% calcd. for CH_3OH); decomposition began at 149 °C and finished at 527 °C with a weight loss of 77.3%; endothermic peak at 182 °C and exothermic peaks at 329 and 462 °C; total weight loss of 79.3% (calc. to PtO residue: 80.1%).

3: Yield: 73%. Anal. Calcd for $C_{34}H_{36}N_{10}Cl_4O_8Pt{\cdot}\frac{1}{2}H_2O$: C, 38.6; N, 13.2; H, 3.5. Found: C, 39.0; N, 13.0; H, 3.3%. IR, cm^{-1}: 3513w, 3464m $\nu(O\text{–}H)_{aliph}$, 3356m ν(N–H), 3129m $\nu(C\text{–}H)_{ar}$, 2946m $\nu(C\text{–}H)_{aliph}$, 1616s $\nu(C{=}N)_{ar}$, 1182w $(C\text{–}Cl)_{ar}$, 1116m, 1053m $\nu(C\text{–}O)_{aliph}$, 510s ν(Pt–N), 330m ν(Pt–Cl). ^{1}H-NMR, δ ppm, *J* Hz, ($\Delta\delta$): 9.35, 1H, *s*, HC^{8} (0.88); 8.98, 1H, *t*, 6.3, HN^{6} (0.60); 8.44, 1H, *s*, HC^{2} (0.17); 7.67, 1H, *d*, 7.8, HC^{12} (0.20); 7.50, 1H, *d*, 8.1, HC^{15} (0.03); 7.32, 1H, *tt*, 7.7, 1.3, HC^{13} (0.01); 7.27, 1H, *t*, 7.7, HC^{14} (-0.04); 6.19, 1H, *d*, 6.0, HC^{16} (0.11); 5.82, 1H, *d*, 6.4, $HO^{17'}$ (0.10); 5.49, 1H, *t*, 4.7, $HO^{20'}$ (-0.16); 5.35, 1H, *d*, 4.7, $HO^{18'}$ (0.04); 5.10, 2H, *d*, 6.3, HC^{9} (0.14); 4.82, 1H, *q*, 5.2, HC^{17} (-0.05); 4.39, 1H, *q*, 4.0, HC^{18} (0.00); 4.19, 1H, *q*, 3.7, HC^{19} (0.03); 3.88, 1H, *m*, H_aC^{20} (0.05); 3.77, 1H,

m, H_bC^{20} (0.04). ^{13}C-NMR, δ ppm, ($\Delta\delta$): 153.90 (C2, 1.37); 152.93 (C6, -2.27); 148.18 (C4, -0.80); 143.59 (C8, 2.96); 136.26 (C10, -0.95); 132.73 (C11, 0.25); 129.53 (C15, 0.16); 128.92 (C12, 0.32); 128.81 (C13, 0.24); 127.30 (C14, 0.07); 116.73 (C5, -4.01); 90.02 (C16, 0.70); 87.23 (C19, 0.19); 74.92 (C17, 0.49); 71.10 (C18, -0.64); 61.96 (C20, -0.57); 42.47 (C9, 0.93). ^{15}N-NMR, δ ppm, (*J* Hz), ($\Delta\delta$): $92.02^{8.98,\ (86.0)\ HN6;\ 5.10,\ HC9}$ N6, (6.57); $135.10^{9.35,\ (5.3)\ HC8}$ N7, (-106.00); $177.37^{9.35,\ (4.1)\ HC8;\ 6.19,\ HC16;\ 4.82,\ HC17}$ N9, (4.96); $225.84^{8.44,\ (14.7)\ HC2}$ N3, (0.82); $236.30^{8.98,\ HN6;\ 8.44,\ (14.7)\ HC2}$ N1, (3.24). ^{195}Pt-NMR, δ (ppm): -2074.81. ESI– MS (methanol, *m/z*): 1083.7 (calc. 1084.1; 100%) $[PtCl_3(L_3)_2]^-$, 1047.8 (calc. 1048.1; 40%) $[PtCl_2(L_3)_2\text{-}H]^-$, 915.8 (calc. 916.1; 80%) $[PtCl_2(L_3)(L_3`)\text{-}H]^-$, 878.0 (calc. 878.1; 10%) $[PtCl(L_3)(L_3`)\text{-}2H]^-$, 486.8 (calc. 487.0; 5%) $[PtCl(L_3`)\text{-}2H]^-$. TG/DTA data: weight loss of 0.9% found between 28–94 °C (0.9% calcd. for $0.5H_2O$); decomposition began at 180 °C and finished at 532 °C with a weight loss of 82.0%; endothermic peak at 211 °C and exothermic peaks at 335, 476 and 492 °C; total weight loss of 82.9% (calc. to PtO residue: 80.1%).

4: Yield: 74%. Anal. Calcd for $C_{34}H_{36}N_{10}Cl_4O_8Pt{\cdot}CH_3OH$: C, 38.9; N, 13.0; H, 3.7. Found: C, 38.7; N, 13.5; H, 3.5%. IR, cm^{-1}: 3442m $\nu(O\text{–}H)_{aliph}$, 3339m ν(N–H), 3130m $\nu(C\text{–}H)_{ar}$, 2937m $\nu(C\text{–}H)_{aliph}$, 1612s $\nu(C{=}N)_{ar}$, 1152w $(C\text{–}Cl)_{ar}$, 1087m, 1056m $\nu(C\text{–}O)_{aliph}$, 524s ν(Pt–N), 335m ν(Pt–Cl). ^{1}H-NMR, δ ppm, *J* Hz, ($\Delta\delta$): 9.35, 1H, *s*, HC^8 (0.92); 8.93, 1H, *t*, 6.3, HN^6 (0.51); 8.46, 1H, *s*, HC^2 (0.19); 7.59, 2H, *d*, 7.7, $HC^{11,15}$ (0.10); 7.37, 2H, *d*, 7.7, $HC^{12,14}$ (-0.02); 6.21, 1H, *d*, 5.4, HC^{16} (0.16); 5.86, 1H, *d*, 5.5, $HO^{17'}$ (0.24); 5.57, 1H, *t*, 5.1, $HO^{20'}$ (-0.03); 5.38, 1H, *d*, 3.7, $HO^{18'}$ (0.12); 4.98, 2H, *d*, 5.7, HC^9 (0.13); 4.82, 1H, *q*, 4.6, HC^{17} (-0.03); 4.42, 1H, *q*, 4.0, HC^{18} (0.06); 4.21, 1H, *m*, HC^{19} (0.07); 3.92, 1H, *m*, H_aC^{20} (0.09); 3.82, 1H, *m*, H_bC^{20} (0.11). ^{13}C-NMR, δ ppm, ($\Delta\delta$): 153.82 (C2, 1.27); 152.80 (C6, -2.37); 147.94 (C4, -0.98); 143.40 (C8, 2.88); 138.11 (C10, -1.48); 132.13 (C13, 0.20); 129.37 (C12, 14, -0.13); 128.45 (C11, 15, -0.01); 116.59 (C5, -4.14); 90.03 (C16, 0.68); 87.06 (C19, -0.03); 74.93 (C17, 0.51); 70.94 (C18, -0.84); 61.80 (C20, -0.78); 43.83 (C9, 0.92). ^{15}N-NMR, δ ppm, (*J* Hz), ($\Delta\delta$): $94.47^{8.93,\ (92.6)\ HN6;\ 4.98,\ (4.7)\ HC9}$ N6, (4.96); $134.42^{9.35,\ (5.8)\ HC8}$ N7, (-106.69); $177.06^{9.35,\ (7.5)\ HC8;\ 6.21,\ (4.0)\ HC16;\ 4.82,\ (3.4)\ HC17}$ N9, (5.45); $225.46^{9.35,\ HC8;\ 8.46,\ (15.4)\ HC2}$ N3, (1.55); $236.07^{8.93,\ HN6}$ N1, (3.61). ^{195}Pt-NMR, δ (ppm): -2071.38. ESI– MS (methanol, *m/z*): 1084.0 (calc. 1084.1; 100%) $[PtCl_3(L_4)_2]^-$, 1047.8 (calc. 1048.1; 50%) $[PtCl_2(L_4)_2\text{-}H]^-$, 915.9 (calc. 916.1; 70%) $[PtCl_2(L_4)(L_4`)\text{-}H]^-$, 878.0 (calc. 878.1; 5%) $[PtCl(L_4)(L_4`)\text{-}2H]^-$, 487.1 (calc. 487.0; 5%) $[PtCl(L_4`)\text{-}2H]^-$. TG/DTA data: weight loss of 2.8% found between 29–137 °C (3.0% calcd. for CH_3tOH); decomposition began at 163 °C and finished at 554 °C with a weight loss of 77.5%; endothermic peak at 179 °C and exothermic peaks at 206, 354 and 502 °C; total weight loss of 80.4% (calc. to PtO residue: 80.5%).

5: Yield: 75%. Anal. Calcd for $C_{34}H_{38}N_{10}Cl_2O_{10}Pt{\cdot}½CH_3OH$: C, 40.3; N, 13.6; H, 3.9. Found: C, 40.5; N, 13.5; H, 3.8%. IR, cm^{-1}: 3584m, 3420m $\nu(O\text{–}H)_{aliph}$, 3343m ν(N–H), 3121m $\nu(C\text{–}H)_{ar}$, 2938m $\nu(C\text{–}H)_{aliph}$, 1613s $\nu(C{=}N)_{ar}$, 1219m $(C\text{–}O)_{ar}$, 1089m, 1057m $\nu(C\text{–}O)_{aliph}$, 545m ν(Pt–N), 326m ν(Pt–Cl). ^{1}H-NMR, δ ppm, *J* Hz, ($\Delta\delta$): 10.08, 1H, s, $HO^{11'}$ (-0.32); 9.17, 1H, *s*, HC^8 (0.72); 9.00, 1H, *t*, 6.1, HN^6 (0.69); 8.45, 1H, *s*, HC^2 (0.12); 7.47, 1H, *dd*, 7.6, 1.2, HC^{15} (0.15); 7.11, 1H, *tt*, 7.6, 1.2, HC^{13} (-0.02); 6.95, 1H, *d*, 8.1, HC^{12} (0.03); 6.74, 1H, *t*, 7.5, HC^{14} (-0.05); 6.19, 1H, *d*, 5.6, HC^{16} (0.11); 5.84, 1H, *d*, 5.6, $HO^{17'}$ (0.14); 5.59, 1H, *t*, 5.8, $HO^{20'}$ (-0.09); 5.35, 1H, *d*, 4.8, $HO^{18'}$ (0.05); 4.99, 2H, *d*, 6.1, HC^9 (0.18); 4.85, 1H, *q*, 5.5, HC^{17} (0.00); 4.40, 1H, *q*, 4.2, HC^{18} (0.01); 4.19, 1H, *q*, 3.3, HC^{19} (0.03); 3.90, 1H, *m*, H_aC^{20} (0.05); 3.78, 1H, *m*, H_bC^{20} (0.06). ^{13}C-NMR, δ ppm, ($\Delta\delta$): 155.86 (C11, 0.02); 153.81

(C2, 1.61); 152.96 (C6, -1.91); 147.86 (C4, -0.82); 143.16 (C8, 2.62); 128.67 (C15, -0.79); 128.38 (C13, -0.06); 124.94 (C10, -1.04); 119.14 (C14, -0.13); 116.67 (C5, -3.90); 115.34 (C12, -0.77); 90.24 (C16, 0.97); 87.22 (C19, 0.26); 74.75 (C17, 0.31); 71.11 (C18, -0.55); 61.98 (C20, -0.47); 40.23 (C9, 0.57). ^{15}N-NMR, δ ppm, (J Hz), ($\Delta\delta$): $95.59^{9.00,\ (93.9)\ HN6;\ 4.99,\ (4.4)\ HC9}$ N6, (5.05); $134.94^{9.17,\ (4.8)\ HC8}$ N7, (-105.70); $177.18^{9.17,\ (4.2)\ HC8;\ 8.45,\ HC2;\ 6.19,\ (4.7)\ HC16;\ 4.85,\ (3.8)\ HC17}$ N9, (5.09); $224.34^{9.17,\ HC8;\ 8.45,\ (15.4)\ HC2}$ N3, (0.70); $234.20^{9.00,\ HN6;\ 8.45,\ (15.7)\ HC2}$ N1, (7.11). ^{195}Pt-NMR, δ (ppm): -2076.80. ESI– MS (methanol, *m/z*): 1045.7 (calc. 1046.2; 45%) $[PtCl_3(L_5)_2]^-$, 1009.7 (calc. 1010.2; 100%) $[PtCl_2(L_5)_2\text{-}H]^-$, 877.8 (calc. 878.1; 50%) $[PtCl_2(L_5)(L_5`)\text{-}H]^-$, 842.0 (calc. 842.2; 55%) $[PtCl(L_5)(L_5`)\text{-}2H]^-$. TG/DTA data: weight loss of 1.4% found between 29–105 °C (1.6% calcd. for 0.5CH_3OH); decomposition began at 186 °C and finished at 483 °C with a weight loss of 77.4%; endothermic peak at 221 °C and exothermic peaks at 228 and 459 °C; total weight loss of 78.7% (calc. to PtO residue: 79.5%).

6: Yield: 70%. Anal. Calcd for $C_{34}H_{38}N_{10}Cl_2O_{10}Pt{\cdot}CH_3OH$: C, 40.2; N, 13.4; H, 4.1. Found: C, 40.4; N, 13.8; H, 3.9%. IR, cm^{-1}: 3319m ν(N–H), 3126m $\nu(C\text{–}H)_{ar}$, 2935m $\nu(C\text{–}H)_{aliph}$, 1613s $\nu(C{=}N)_{ar}$, 1215m $(C\text{–}O)_{ar}$, 1080m, 1056m $\nu(C\text{–}O)_{aliph}$, 527s ν(Pt–N), 333m ν(Pt–Cl). ^{1}H-NMR, δ ppm, J Hz, ($\Delta\delta$): 9.57, 1H, s, $HO^{12'}$ (0.09); 9.26, 1H, *s*, HC^{8} (0.84); 8.88, 1H, *t*, 6.1, HN^{6} (0.59); 8.45, 1H, *s*, HC^{2} (0.18); 7.13, 1H, *t*, 7.8, HC^{14} (-0.01); 7.01, 1H, *d*, 2.0, HC^{11} (0.06); 6.99, 1H, *d*, 7.8, HC^{15} (0.11); 6.75, 1H, *dd*, 8.0, 2.2, HC^{13} (0.03); 6.20, 1H, *d*, 5.6, HC^{16} (0.14); 5.85, 1H, *d*, 5.6, $HO^{17'}$ (0.08); 5.56, 1H, *t*, 5.5, $HO^{20'}$ (-0.07); 5.37, 1H, *d*, 3.8, $HO^{18'}$ (0.09); 4.89, 2H, *d*, 6.0, HC^{9} (0.07); 4.84, 1H, *q*, 4.9, HC^{17} (-0.02); 4.41, 1H, *q*, 3.8, HC^{18} (0.04); 4.20, 1H, *q*, 3.4, HC^{19} (0.05); 3.91, 1H, *m*, H_aC^{20} (0.08); 3.80, 1H, *m*, H_bC^{20} (0.08). ^{13}C-NMR, δ ppm, ($\Delta\delta$): 158.18 (C12, 0.05); 153.83 (C2, 1.31); 152.96 (C6, -2.29); 147.90 (C4, -0.88); 143.33 (C8, 2.93); 140.48 (C10, -1.44); 129.45 (C14, 0.11); 118.01 (C15, -0.14); 116.59 (C5, -4.07); 114.47 (C11, 0.07); 114.14 (C13, 0.29); 90.13 (C16, 0.76); 87.18 (C19, 0.11); 74.81 (C17, 0.46); 71.05 (C18, -0.73); 61.91 (C20, -0.66); 44.41 (C9, 1.06). ^{15}N-NMR, δ ppm, (J Hz), ($\Delta\delta$): $95.32^{8.88,\ (91.9)\ HN6;\ 4.89,\ HC9}$ N6, (5.51); $134.73^{9.26,\ HC8}$ N7, (-105.78); $177.10^{9.26,\ HC8;\ 6.21,\ HC16;\ 4.84,\ HC17}$ N9, (6.21); $225.38^{9.26,\ HC8;\ 8.45,\ (14.8)\ HC2}$ N3, (1.91); $236.21^{8.88,\ HN6}$ N1, (3.85). ^{195}Pt-NMR, δ (ppm): -2074.68. ESI– MS (methanol, *m/z*): 1046.2 (calc. 1046.2; 95%) $[PtCl_3(L_6)_2]^-$, 1010.1 (calc. 1010.2; 100%) $[PtCl_2(L_6)_2\text{-}H]^-$, 878.2 (calc. 878.1; 55%) $[PtCl_2(L_6)(L_6`)\text{-}H]^-$, 842.7 (calc. 842.2; 5%) $[PtCl(L_6)(L_6`)\text{-}2H]^-$, 469.0 (calc. 469.0; 5%) $[PtCl(L_6`)\text{-}2H]^-$. TG/DTA data: weight loss of 3.2% found between 29–116 °C (3.1% calcd. for CH_3OH); decomposition began at 116 °C and finished at 539 °C with a weight loss of 77.2%; endothermic peak at 57 °C and exothermic peaks at 169 and 486 °C; total weight loss of 80.4% (calc. to PtO residue: 79.8%).

7: Yield: 68%. Anal. Calcd for $C_{36}H_{42}N_{10}Cl_2O_{12}Pt{\cdot}CH_3OH$: C, 40.2; N, 12.7; H, 4.2. Found: C, 40.5; N, 13.2; H, 4.0%. IR, cm^{-1}: 3433m $\nu(O\text{–}H)_{aliph}$, 3340m ν(N–H), 3127m $\nu(C\text{–}H)_{ar}$, 2937m $\nu(C\text{–}H)_{aliph}$, 1615s $\nu(C{=}N)_{ar}$, 1226m $(C\text{–}O)_{ar}$, 1080m, 1056m $\nu(C\text{–}O)_{aliph}$, 530s ν(Pt–N), 340m ν(Pt–Cl). ^{1}H-NMR, δ ppm, J Hz, ($\Delta\delta$): 9.25, 1H, *s*, $HO^{11'}$ (-0.39); 9.13, 1H, *s*, HC^{8} (0.69); 8.97, 1H, *t*, 6.0, HN^{6} (0.75); 8.46, 1H, *s*, HC^{2} (0.15); 7.11, 1H, *dd*, 7.6, 1.2, HC^{15} (0.19); 6.93, 1H, *dd*, 8.2, 1.2, HC^{13} (0.03); 6.74, 1H, *t*, 8.0, HC^{14} (0.00); 6.19, 1H, *d*, 5.8, HC^{16} (0.13); 5.86, 1H, *d*, 5.6, $HO^{17'}$ (0.19); 5.62, 1H, *t*, 6.2, $HO^{20'}$ (-0.02); 5.38, 1H, *d*, 4.1, $HO^{18'}$ (0.12); 5.01, 2H, *d*, 6.0, HC^{9} (0.18); 4.90, 1H, *q*, 5.0, HC^{17} (0.06); 4.42, 1H, *q*, 3.7, HC^{18} (0.06); 4.21, 1H, *q*, 3.1, HC^{19} (0.07); 3.90, 1H, *m*, H_aC^{20} (0.05); 3.87, 3H, *s*, $HC^{12'}$ (0.04); 3.80, 1H, *m*, H_bC^{20} (0.08). ^{13}C-NMR, δ ppm, ($\Delta\delta$): 153.80 (C2, 1.49); 152.92 (C6, -4.06);

147.99 (C12, -0.40); 147.82 (C4, -0.92); 144.83 (C11, -0.14); 143.29 (C8, 2.73); 125.25 (C10, -1.21); 120.64 (C15, -0.43); 119.00 (C14, 0.04); 116.68 (C5, -3.97); 110.97 (C13, -0.08); 90.37 (C16, 1.05); 87.33 (C19, 0.31); 74.56 (C17, 0.10); 71.23 (C18, -0.49); 62.07 (C20, -0.44); 55.83 (C12′, -0.74); 40.09 (C9, 0.67). ^{15}N-NMR, *δ* ppm, (*J* Hz), (Δ*δ*): $96.41^{8.97,\ (92.7)\ HN6;\ 5.01,\ HC9}$ N6, (5.82); $134.96^{9.13,\ HC8}$ N7, (-106.22); $176.91^{9.13,\ HC8;\ 6.19,\ HC16;\ 4.90,\ HC17}$ N9, (4.78); $223.96^{9.13,\ HC8;\ 8.46,\ (15.5)\ HC2}$ N3, (0.34); $234.73^{8.97,\ HN6;\ 8.46,\ (15.6)\ HC2}$ N1, (6.98). ^{195}Pt-NMR, *δ* (ppm): -2078.30. ESI– MS (methanol, *m/z*): 1106.1 (calc. 1106.2; 75%) $[PtCl_3(L_7)_2]^-$, 1070.2 (calc. 1070.2; 100%) $[PtCl_2(L_7)_2\text{-}H]^-$, 938.2 (calc. 938.2; 45%) $[PtCl_2(L_7)(L_7`)\text{-}H]^-$, 902.3 (calc. 902.2; 15%) $[PtCl(L_7`)\text{-}2H]^-$. TG/DTA data: weight loss of 2.5% found between 31–107 °C (2.9% calcd. for CH_3OH); decomposition began at 107 °C and finished at 476 °C with a weight loss of 76.2%; endothermic peaks at 54 and 191 °C and exothermic peaks at 313 and 451 °C; total weight loss of 78.7% (calc. to PtO residue: 80.9%).

8: Yield: 75%. Anal. Calcd for $C_{34}H_{36}N_{10}Cl_2F_2O_8Pt$·½$CH_3OH$: C, 40.1; N, 13.6; H, 3.7. Found: C, 40.1; N, 13.8; H, 3.5%. IR, cm^{-1}: 3342m ν(N–H), 3133m ν(C–H)$_{ar}$, 2932m ν(C–H)$_{aliph}$, 1613s ν(C=N)$_{ar}$, 1215m (C–F)$_{ar}$, 1080m, 1054m ν(C–O)$_{aliph}$, 520s ν(Pt–N), 331m ν(Pt–Cl). ^{1}H-NMR, *δ* ppm, *J* Hz, (Δ*δ*): 9.33, 1H, *s*, HC^8 (0.90); 8.91, 1H, *t*, 5.8, HN^6 (0.52); 8.47, 1H, *s*, HC^2 (0.19); 7.61, 2H, *m*, HC^{11,15} (0.09); 7.13, 2H, *tt*, 8.5, 1.8, HC^{12,14} (-0.02); 6.21, 1H, *d*, 5.5, HC^{16} (0.15); 5.85, 1H, *br*, HO$^{17′}$ (0.12); 5.56, 1H, *br*, HO$^{20′}$ (-0.06); 5.37, 1H, *br*, HO$^{18′}$ (0.10); 4.97, 2H, *d*, 6.0, HC^9 (0.11); 4.82, 1H, *t*, 5.4, HC^{17} (-0.04); 4.41, 1H, *t*, 4.1, HC^{18} (0.04); 4.21, 1H, *q*, 3.3, HC^{19} (0.06); 3.92, 1H, *m*, H$_a$C^{20} (0.09); 3.81, 1H, *m*, H$_b$C^{20} (0.09). ^{13}C-NMR, *δ* ppm, (Δ*δ*): 163.15, 160.73 (C13, 0.07); 153.85 (C2, 1.31); 152.84 (C6, -2.29); 147.96 (C4, -0.93); 143.39 (C8, 2.90); 135.19, 135.16 (C10, -1.49); 129.67, 129.59 (C11, 15, -0.02); 116.61 (C5, -4.13); 115.25, 115.03 (C12, 14, 0.07); 90.06 (C16, 0.69); 87.11 (C19, 0.03); 74.98 (C17, 0.58); 70.99 (C18, -0.79); 61.84 (C20, -0.74); 43.83 (C9, 1.01). ^{15}N-NMR, *δ* ppm, (*J* Hz), (Δ*δ*): $97.19^{8.91,\ (92.2)\ HN6;\ 4.97,\ HC9}$ N6, (6.52); $135.61^{9.33,\ HC8}$ N7, (-105.37); $177.98^{9.33,\ HC8;\ 6.21,\ HC16}$ N9, (6.32); $225.11^{9.33,\ HC8;\ 8.47,\ (15.1)\ HC2}$ N3, (0.33); $236.22^{8.91,\ HN6}$ N1, (3.67). ^{195}Pt-NMR, *δ* (ppm): -2071.68. ESI– MS (methanol, *m/z*): 1050.1 (calc. 1050.1; 100%) $[PtCl_3(L_8)_2]^-$, 1013.7 (calc. 1014.2; 25%) $[PtCl_2(L_8)_2\text{-}H]^-$, 882.0 (calc. 882.1; 30%) $[PtCl_2(L_8)(L_8`)\text{-}H]^-$, 845.9 (calc. 846.1; 5%) $[PtCl(L_8`)\text{-}2H]^-$. TG/DTA data: weight loss of 1.5% found between 31–103 °C (1.5% calcd. for 0.5CH_3OH); decomposition began at 162 °C and finished at 513 °C with a weight loss of 79.4%; endothermic peaks at 49 and 188 °C and exothermic peak at 481 °C; total weight loss of 80.9% (calc. to PtO residue: 79.6%).

9: Yield: 74%. Anal. Calcd for $C_{36}H_{42}N_{10}Cl_2O_8Pt$·½$CH_3OH$: C, 42.8; N, 13.7; H, 4.3. Found: C, 43.0; N, 13.7; H, 4.2%. IR, cm^{-1}: 3516m ν(O–H)$_{aliph}$, 3358m ν(N–H), 3127m ν(C–H)$_{ar}$, 2923m ν(C–H)$_{aliph}$, 1614s ν(C=N)$_{ar}$, 1081m, 1057m ν(C–O)$_{aliph}$, 516s ν(Pt–N), 332m ν(Pt–Cl). ^{1}H-NMR, *δ* ppm, *J* Hz, (Δ*δ*): 9.24, 1H, *s*, HC^8 (0.83); 8.86, 1H, *t*, 6.0, HN^6 (0.55); 8.47, 1H, *s*, HC^2 (0.19); 7.45, 2H, *d*, 7.9, HC^{11,15} (0.11); 7.11, 2H, *d*, 7.8, HC^{12,14} (-0.03); 6.21, 1H, *d*, 5.6, HC^{16} (0.15); 5.84, 1H, *d*, 6.5, HO$^{17′}$ (0.08); 5.51, 1H, *m*, HO$^{20′}$ (-0.25); 5.37, 1H, *d*, 4.7, HO$^{18′}$ (0.09); 4.88, 2H, *d*, 5.9, HC^9 (0.05); 4.84, 1H, *q*, 5.9, HC^{17} (-0.02); 4.40, 1H, *q*, 4.2, HC^{18} (0.03); 4.19, 1H, *q*, 3.1, HC^{19} (0.04); 3.90, 1H, *m*, H$_a$C^{20} (0.07); 3.79, 1H, *m*, H$_b$C^{20} (0.07); 2.27, 3H, s, HC$^{13′}$ (-0.01). ^{13}C-NMR, *δ* ppm, (Δ*δ*): 153.88 (C2, 1.34); 152.91 (C6, -2.32); 147.90 (C4, -0.88); 143.29 (C8, 2.89); 136.64 (C10, -0.74); 135.76 (C13, -0.51); 129.13 (C12, 14, 0.11); 127.60 (C11, 15, -0.03); 116.62 (C5, -4.07); 90.14 (C16, 0.75);

87.19 (C19, 0.10); 74.92 (C17, 0.54); 71.07 (C18, -0.72); 61.92 (C20, -0.67); 44.36 (C9, 1.12); 20.45 (C13′, 0.02). ^{15}N-NMR, δ ppm, (J Hz), ($\Delta\delta$): 97.19$^{8.86,\ (92.1)\ HN6;\ 4.88,\ HC9}$ N6, (4.89); 134.96$^{9.24,\ (5.5)\ HC8}$ N7, (-106.10); 177.21$^{9.24,\ (5.0)\ HC8;\ 8.47,\ HC2;\ 6.21,\ (4.8)\ HC16;\ 4.84,\ HC17}$ N9, (5.86); 225.22$^{9.24,\ HC8;\ 8.47,\ (14.5)\ HC2}$ N3, (1.76); 236.74$^{8.86,\ HN6}$ N1, (4.46). ^{195}Pt-NMR, δ (ppm): -2073.15. ESI– MS (methanol, *m/z*): 1041.9 (calc. 1042.2; 100%) $[PtCl_3(L_9)_2]^-$, 1005.8 (calc. 1006.2; 55%) $[PtCl_2(L_9)_2\text{-}H]^-$, 873.9 (calc. 874.2; 70%) $[PtCl_2(L_9)(L_9`)\text{-}H]^-$, 838.1 (calc. 838.2; 20%) $[PtCl(L_9)(L_9`)\text{-}2H]^-$. TG/DTA data: weight loss of 1.8% found between 28–140 °C (1.6% calcd. for $0.5CH_3OH$); decomposition began at 173 °C and finished at 521 °C with a weight loss of 78.2%; endothermic peak at 202 °C and exothermic peak at 478 °C; total weight loss of 80.1% (calc. to PtO residue: 79.4%).

10: Yield: 68%. Anal. Calcd for $C_{34}H_{36}N_{10}Cl_4O_{10}Pt{\cdot}¾CH_3OH$: C, 37.8; N, 12.7; H, 3.6. Found: C, 37.8; N, 13.2; H, 3.2%. IR, cm^{-1}: 3337m ν(N–H), 3123m ν(C–H)$_{ar}$, 2946m ν(C–H)$_{aliph}$, 1605s ν(C=N)$_{ar}$, 1215m (C–O)$_{ar}$, 1080m, 1061m ν(C–O)$_{aliph}$, 525s ν(Pt–N), 329s ν(Pt–Cl). ^{1}H-NMR, δ ppm, J Hz, ($\Delta\delta$): 9.61, 1H, s, HO$^{12′}$ (0.08); 9.30, 1H, *s*, HC^8 (0.83); 9.03, 1H, *t*, 6.0, HN^6 (0.26); 7.14, 1H, *t*, 7.8, HC^{14} (-0.02); 7.02, 1H, *s*, HC^{11} (0.08); 7.00, 1H, *d*, 7.8, HC^{15} (0.12); 6.77, 1H, *dd*, 8.0, 1.6, HC^{13} (0.03); 6.17, 1H, *d*, 4.9, HC^{16} (0.15); 5.93, 1H, *d*, 5.4, HO$^{17′}$ (0.25); 5.45, 1H, *t*, 5.4, HO$^{20′}$ (0.18); 5.42, 1H, *d*, 4.0, HO$^{18′}$ (0.15); 4.84, 2H, *d*, 5.4, HC^9 (0.08); 4.77, 1H, *q*, 4.8, HC^{17} (0.01); 4.44, 1H, *q*, 4.3, HC^{18} (-0.03); 4.23, 1H, *q*, 3.5, HC^{19} (0.10); 3.95, 1H, *m*, H$_a$C^{20} (0.11); 3.84, 1H, *m*, H$_b$C^{20} (0.09). ^{13}C-NMR, δ ppm, ($\Delta\delta$): 158.05 (C12, -0.09); 154.92 (C6, -0.66); 153.25 (C2, -0.53); 148.91 (C4, -1.19); 143.46 (C8, 3.03); 139.41 (C10, -1.65); 129.35 (C14, -0.06); 118.09 (C15, -0.13); 115.52 (C5, -3.76); 114.48 (C11, 0.04); 114.23 (C13, 0.21); 89.75 (C16, 1.15); 86.78 (C19, 0.09); 75.03 (C17, 0.40); 70.59 (C18, -0.79); 61.44 (C20, -0.76); 44.67 (C9, 1.12). ^{15}N-NMR, δ ppm, (J Hz), ($\Delta\delta$): 100.93$^{9.03,\ (93.9)\ HN6;\ 4.84,\ HC9}$ N6, (6.15); 134.66$^{9.30,\ HC8}$ N7, (-106.52); 177.14$^{9.30,\ HC8;\ 6.17,\ HC16;\ 4.77,\ HC17}$ N9, (5.02); 220.87$^{9.30,\ HC8}$ N3, (-0.31); 230.87$^{9.03,\ HN6}$ N1, (3.13). ^{195}Pt-NMR, δ (ppm): -2084.94. ESI– MS (methanol, *m/z*): 1115.8 (calc. 1116.1; 95%) $[PtCl_3(L_{10})_2]^-$, 1079.9 (calc. 1080.1; 90%) $[PtCl_2(L_{10})_2\text{-}H]^-$, 948.1 (calc. 948.1; 100%) $[PtCl_2(L_{10})(L_{10}`)\text{-}H]^-$, 910.0 (calc. 910.1; 15%) $[PtCl(L_{10})(L_{10}`)\text{-}2H]^-$. TG/DTA data: weight loss of 2.1% found between 31–128 °C (2.2% calcd. for $0.75CH_3OH$); decomposition began at 150 °C and finished at 563 °C with a weight loss of 77.3%; endothermic peaks at 54, 91 and 193 °C and exothermic peaks at 215 and 460 °C; total weight loss of 79.4% (calc. to PtO residue: 80.9%).

11: Yield: 69%. Anal. Calcd for $C_{34}H_{36}N_{10}Cl_4O_{10}Pt{\cdot}CH_3OH$: C, 37.8; N, 12.6; H, 3.6. Found: C, 37.5; N, 13.0; H, 3.5%. IR, cm^{-1}: 3339s ν(N–H), 3127s ν(C–H)$_{ar}$, 2931m ν(C–H)$_{aliph}$, 1609s ν(C=N)$_{ar}$, 1217m (C–O)$_{ar}$, 1173m (C–Cl)$_{ar}$, 1079m, 1060m ν(C–O)$_{aliph}$, 534s ν(Pt–N), 326m ν(Pt–Cl). ^{1}H-NMR, δ ppm, J Hz, ($\Delta\delta$): 9.57, 1H, *s*, HO$^{13′}$ (0.07); 9.28, 1H, *t*, 6.2, HN^6 (0.60); 9.27, 1H, *s*, HC^8 (0.83); 7.39, 2H, *d*, 8.6, HC^{11,15} (0.09); 6.79, 2H, *d*, 8.3, HC^{12,14} (-0.03); 6.15, 1H, *d*, 5.1, HC^{16} (0.14); 5.92, 1H, *d*, 5.6, HO$^{17′}$ (0.24); 5.45, 1H, *t*, 5.3, HO$^{20′}$ (0.16); 5.42, 1H, *d*, 4.8, HO$^{18′}$ (0.13); 4.78, 2H, *d*, 6.1, HC^9 (0.07); 4.74, 1H, *q*, 5.0, HC^{17} (-0.02); 4.41, 1H, *q*, 4.6, HC^{18} (0.04); 4.20, 1H, *q*, 3.4, HC^{19} (0.07); 3.93, 1H, *m*, H$_a$C^{20} (0.09); 3.83, 1H, *m*, H$_b$C^{20} (0.08). ^{13}C-NMR, δ ppm, ($\Delta\delta$): 157.34 (C13, 0.22); 155.04 (C2, 1.30); 153.23 (C6, -2.18); 148.98 (C4, -1.01); 143.46 (C8, 3.14); 129.20 (C11, 15, 0.02); 128.21 (C10, -1.57); 115.57 (C5, -3.68); 115.28 (C12, 14, 0.07); 89.80 (C16, 1.20); 86.87 (C19, 0.22); 75.22 (C17, 0.62); 70.68 (C18, -0.67); 61.51 (C20, -0.67); 44.43 (C9, 1.13). ^{15}N-NMR, δ ppm, (J Hz), ($\Delta\delta$):

101.29$^{9.28,\ (97.2)\ HN6}$ N6, (3.30); 135.35$^{9.27,\ (5.4)\ HC8}$ N7, (-106.24); 177.72$^{9.27,\ (5.2)\ HC8;\ 6.15,\ HC16;\ 4.74,\ HC17}$ N9, (5.00); 221.71$^{9.27,\ HC8}$ N3, (0.26); 231.40$^{9.28,\ HN6}$ N1, (3.45). ^{195}Pt-NMR, δ (ppm): -2083.47. ESI– MS (methanol, *m/z*): 1115.8 (calc. 1116.1; 80%) $[PtCl_3(L_{11})_2]^-$, 1079.8 (calc. 1080.1; 65%) $[PtCl_2(L_{11})_2\text{-}H]^-$, 948.2 (calc. 948.1; 100%) $[PtCl_2(L_{11})(L_{11}`)\text{-}H]^-$; 910.2 (calc. 910.1; 5%) $[PtCl(L_{11})(L_{11}`)\text{-}2H]^-$. TG/DTA data: weight loss of 2.6% found between 31–110 °C (2.9% calcd. for CH_3OH); decomposition began at 147 °C and finished at 500 °C with a weight loss of 78.1%; endothermic peak at 59 °C and exothermic peaks at 183 and 476 °C; total weight loss of 80.7% (calc. to PtO residue: 81.0%).

12: Yield: 71%. Anal. Calcd for $C_{36}H_{40}N_{10}Cl_4O_{12}Pt{\cdot}CH_3OH$: C, 37.9; N, 11.9; H, 3.8. Found: C, 37.8; N, 12.2; H, 3.6%. IR, cm^{-1}: 3417m $\nu(O\text{–}H)_{aliph}$, 3321s ν(N–H), 3127m $\nu(C\text{–}H)_{ar}$, 2938m $\nu(C\text{–}H)_{aliph}$, 1616s $\nu(C{=}N)_{ar}$, 1217m $(C\text{–}O)_{ar}$, 1163w $(C\text{–}Cl)_{ar}$, 1072m, 1058m $\nu(C\text{–}O)_{aliph}$, 530s ν(Pt–N), 339s ν(Pt–Cl). ^{1}H-NMR, δ ppm, *J* Hz, ($\Delta\delta$): 9.31, 1H, *t*, 6.1, HN^6 (0.81); 9.27, 1H, *br*, $HO^{11'}$ (0.21); 9.17, 1H, *s*, HC^8 (0.69); 7.08, 1H, *d*, 8.1, HC^{15} (0.17); 6.93, 1H, *d*, 8.1, HC^{13} (0.04); 6.66, 1H, *t*, 8.0, HC^{14} (-0.09); 6.14, 1H, *d*, 5.2, HC^{16} (0.13); 5.90, 1H, *d*, 5.5, $HO^{17'}$ (0.22); 5.45, 1H, *t*, 5.5, $HO^{20'}$ (0.14); 5.35, 1H, *br*, $HO^{18'}$ (0.08); 4.97, 2H, *br*, HC^9 (0.15); 4.67, 1H, *q*, 4.8, HC^{17} (-0.08); 4.42, 1H, *q*, 4.2, HC^{18} (0.06); 4.19, 1H, *q*, 3.4, HC^{19} (0.07); 3.93, 1H, *m*, H_aC^{20} (0.10); 3.87, 3H, *s*, $HC^{12'}$ (0.03); 3.84, 1H, *m*, H_bC^{20} (0.10). ^{13}C-NMR, δ ppm, ($\Delta\delta$): 155.09 (C2, 1.34); 153.42 (C6, -2.31); 149.16 (C4, -0.90); 147.91 (C12, -0.03); 144.87 (C11, 0.23); 143.56 (C8, 3.04); 124.41 (C10, -1.17); 118.88 (C15, -1.51); 118.66 (C14, -0.23); 115.74 (C5, -3.66); 110.73 (C13, -0.09); 86.72 (C16, -1.94); 86.68 (C19, -0.04); 75.46 (C17, 0.73); 70.51 (C18, -0.91); 61.75 (C20, -0.48); 55.84 (C12′, 0.02); 40.44 (C9, 1.07). ^{15}N-NMR, δ ppm, (*J* Hz), ($\Delta\delta$): 98.36$^{9.31,\ (93.7)\ HN6}$ N6, (4.90); 136.08$^{9.17,\ HC8}$ N7, (-105.72); 177.56$^{9.17,\ HC8;\ 6.14,\ HC16;\ 4.67,\ HC17}$ N9, (5.03); 221.60$^{9.17,\ HC8}$ N3, (0.16); 230.99$^{9.31,\ HN6}$ N1, (3.92). ^{195}Pt-NMR, δ (ppm): -2088.28. ESI– MS (methanol, *m/z*): 1176.1 (calc. 1176.1; 100%) $[PtCl_3(L_{12})_2]^-$, 1140.0 (calc. 1140.1; 20%) $[PtCl_2(L_{12})_2\text{-}H]^-$, 1008.1 (calc. 1008.1; 15%) $[PtCl_2(L_{12})(L_{12}`)\text{-}H]^-$; 970.2 (calc. 970.1; 5%) $[PtCl(L_{12})(L_{12}`)\text{-}H]^-$. TG/DTA data: weight loss of 2.5% found between 27–101 °C (2.7% calcd. for CH_3OH); decomposition began at 131 °C and finished at 564 °C with a weight loss of 78.5%; endothermic peak at 48 °C and exothermic peaks at 150, 193, 378 and 524 °C; total weight loss of 81.1% (calc. to PtO residue: 82.0%).

13: Yield: 65%. Anal. Calcd for $C_{36}H_{40}N_{10}Cl_4O_{10}Pt{\cdot}\frac{3}{4}CH_3OH$: C, 38.9; N, 12.4; H, 3.8. Found: C, 38.6; N, 12.9; H, 3.4%. IR, cm^{-1}: 3408m $\nu(O\text{–}H)_{aliph}$, 3308s ν(N–H), 3129m $\nu(C\text{–}H)_{ar}$, 2927m $\nu(C\text{–}H)_{aliph}$, 1613s $\nu(C{=}N)_{ar}$, 1214m $(C\text{–}O)_{ar}$, 1164w $(C\text{–}Cl)_{ar}$, 1078m, 1055m $\nu(C\text{–}O)_{aliph}$, 533s ν(Pt–N), 334s ν(Pt–Cl). ^{1}H-NMR, δ ppm, *J* Hz, ($\Delta\delta$): 9.73, 1H, *s*, $HO^{11'}$ (0.08); 9.36, 1H, *t*, 6.4, HN^6 (0.85); 9.26, 1H, *br*, HC^8 (0.78); 7.23, 1H, *br*, HC^{15} (0.11); 6.91, 1H, *d*, 8.2, HC^{12} (-0.03); 6.84, 1H, *d*, 7.7, HC^{13} (0.01); 6.03, 1H, *d*, 5.0, HC^{16} (0.01); 5.80, 1H, *d*, 4.8, $HO^{17'}$ (0.09); 5.38, 1H, *d*, 5.1, $HO^{18'}$ (0.06); 5.35, 1H, *br*, $HO^{20'}$ (0.06); 4.85, 2H, *d*, 5.4, HC^9 (0.07); 4.66, 1H, *q*, 5.1, HC^{17} (-0.10); 4.37, 1H, *m*, HC^{18} (0.00); 4.13, 1H, *q*, 3.4, HC^{19} (-0.01); 3.91, 1H, *m*, H_aC^{20} (0.07); 3.83, 1H, *m*, H_bC^{20} (0.08); 2.15, 3H, *s*, $HC^{14'}$ (-0.04). ^{13}C-NMR, δ ppm, ($\Delta\delta$): 154.93 (C6, -0.60); 153.74 (C2, 0.12); 153.23 (C11, -0.09); 149.08 (C4, -0.83); 142.26 (C8, 1.81); 129.24 (C15, -0.05); 128.90 (C12, 0.23); 127.81 (C10, -0.07); 123.88 (C14, -0.92); 116.03 (C5, -3.27); 114.83 (C13, -0.54); 89.98 (C16, 1.37); 86.64 (C19, 0.00); 74.86 (C17, 0.21); 70.47 (C18, -0.85); 61.50 (C20, -0.63); 40.34 (C9, 0.77); 20.01

(C14′, 0.08). ^{15}N-NMR, δ ppm, (J Hz), ($\Delta\delta$): 98.95$^{9.36,\ (93.3)\ HN6}$ N6, (4.96); 135.69$^{9.26,\ HC8}$ N7, (-105.76); 177.64$^{9.26,\ HC8;\ 6.03,\ HC16}$ N9, (5.33); N3 – no detected; 230.93$^{9.36,\ HN6}$ N1, (4.89). ^{195}Pt-NMR, δ (ppm): -2091.62. ESI– MS (methanol, *m/z*): 1144.0 (calc. 1144.1; 100%) $[PtCl_3(L_{13})_2]^-$, 1107.9 (calc. 1108.1; 20%) $[PtCl_2(L_{13})_2\text{-H}]^-$, 976.1 (calc. 976.1; 30%) $[PtCl_2(L_{13})(L_{13}`)\text{-H}]^-$, 938.3 (calc. 938.1; 5%) $[PtCl(L_{13})(L_{13}`)\text{-2H}]^-$. TG/DTA data: weight loss of 2.0% found between 30–86 °C (2.1% calcd. for 0.75CH_3OH); decomposition began at 131 °C and finished at 511 °C with a weight loss of 76.3%; endothermic peak at 54 °C and exothermic peaks at 364 and 467 °C; total weight loss of 78.3 (calc. to PtO residue: 80.5%).

Figure S1. (**a**) The mass spectra of complex **7** dissolved in methanol and measured in the negative ionization mode (*top spectrum*), and interacting systems with L-methionine measured immediately after the preparation (*middle spectrum*) and 12 h after the preparation (*lower spectrum*); (**b**) the mass spectra of complex **12** dissolved in methanol and measured in the positive ionization mode (*top spectrum*), and interacting systems with L-methionine measured immediately after the preparation (*middle spectrum*) and 12 h after the preparation (*lower spectrum*).

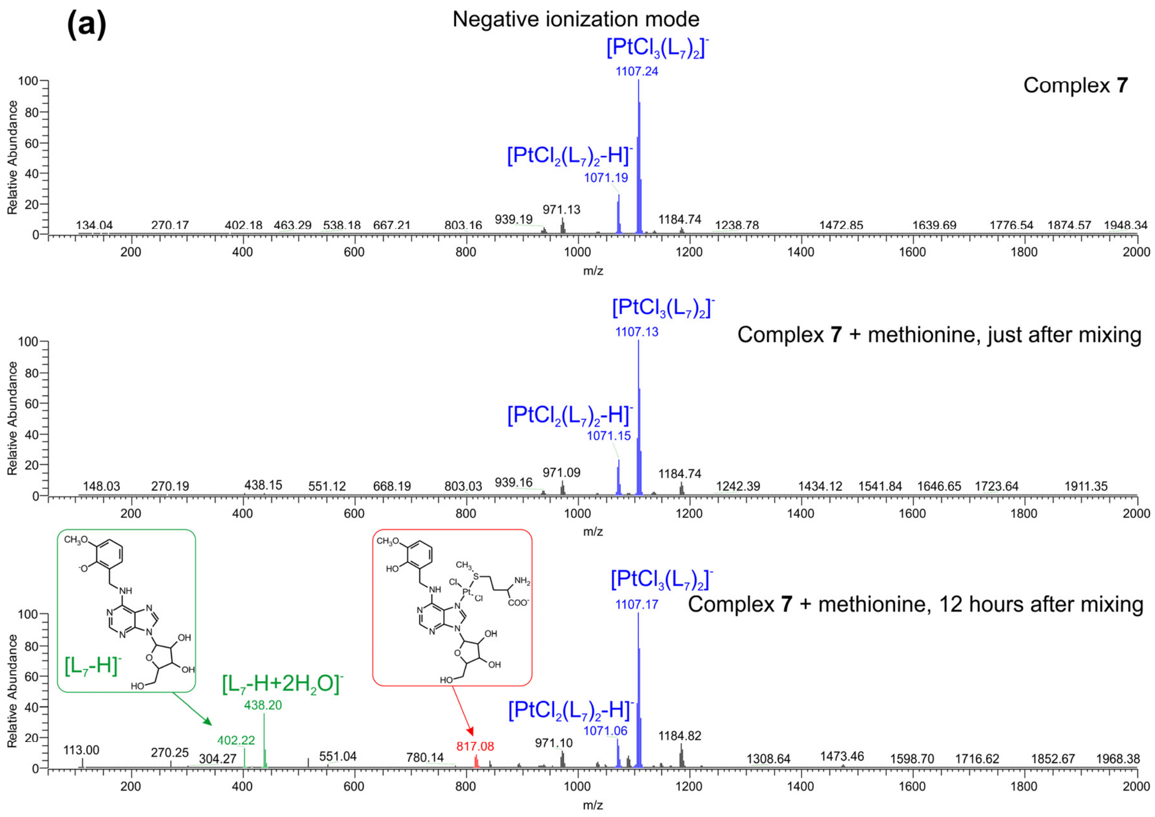

Figure S1. *Cont.*

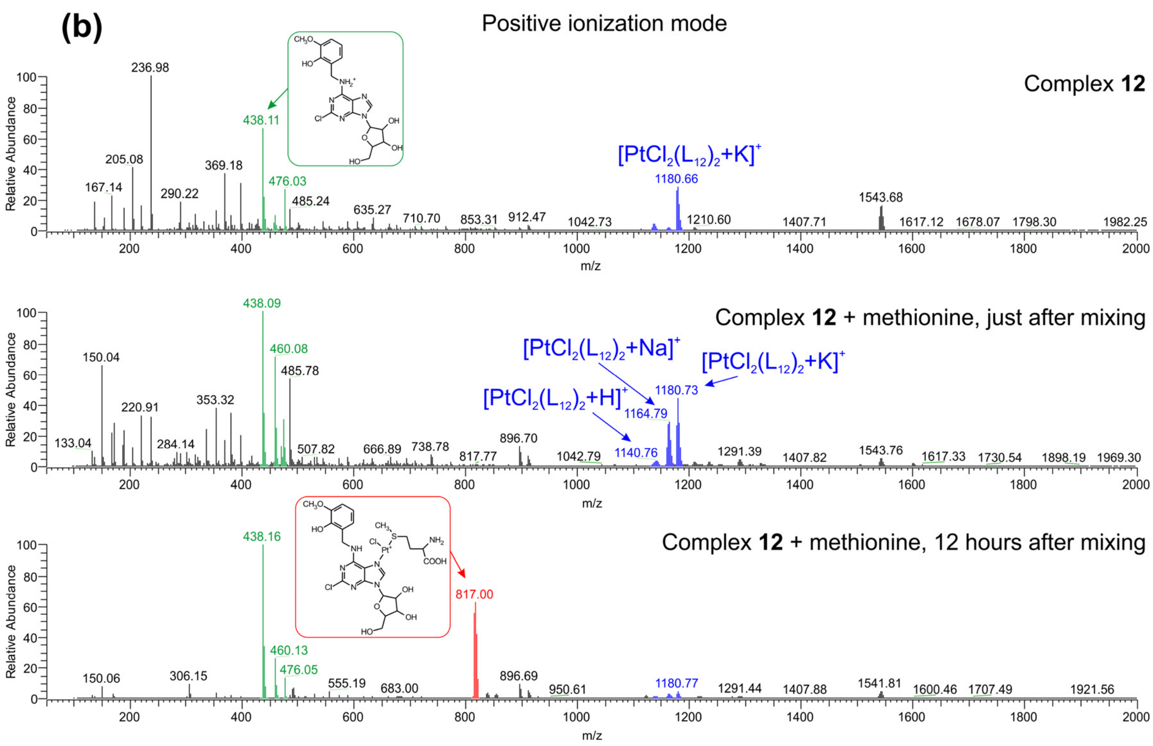

Supplement: Supplementary file 1 [file molecules-18-06990-s001.pdf]
